# Association between the Number of Childbirths and the Progress of Atherosclerosis among Women with Diabetes: A Cohort Study Based on Chinese Population

**DOI:** 10.1155/2019/4874121

**Published:** 2019-01-27

**Authors:** Jie Wang, Yu Pei, Kang Chen, Wenhua Yan, Anping Wang, Yijun Li, Jia Li, Haibing Wang, Ping An, Linxi Zhang, Yingnan Ye, Xinye Jin, Guang Ning, Yiming Mu, Weijun Gu

**Affiliations:** ^1^School of Medicine, Nankai University, Tianjin, China; ^2^Department of Endocrinology, Chinese PLA General Hospital, Beijing, China; ^3^Shanghai National Research Centre for Endocrine and Metabolic Diseases, State Key Laboratory of Medical Genomics, Shanghai Institute for Endocrine and Metabolic Diseases, Ruijin Hospital, Shanghai Jiaotong University School of Medicine, Shanghai, China

## Abstract

**Background:**

The aim of this study is to explore the association between the number of childbirths and the progress of atherosclerosis among Chinese women with hypertension or diabetes.

**Methods:**

In total, 1159 Chinese parous women from a community longitudinal survey conducted in the communities of Shijingshan district, Beijing, China, were included in our study. They were divided into three groups according to the number of childbirths, and the change in pulse wave velocity (PWV) was as an indicator of the progression of atherosclerosis because the increased PWV reflected the more serious atherosclerosis. After 3 years, we conducted follow-up visits to the subjects. Logistical regression analyses were applied to investigate the relationship between the number of childbirths and the progression of atherosclerotic stiffness and a stratification analysis was performed for history of hypertension and diabetes.

**Results:**

After 3-year follow-up, among women with diabetes, the OR of women with 2 childbirths was significant [3.5 (95% confidence interval 1.5, 7.9)] in model I, [3.1 (95% confidence interval 1.3, 7.2)] in model II, and the OR of women with ≥3 childbirths was significant [4.4 (95% confidence interval 1.3, 14.5)] in model I, [4.1 (95% confidence interval 1.2, 14.3)] in model II. Among women with hypertension, the risk of the progress of atherosclerosis was not significant.

**Conclusion:**

The increasing number of childbirths is associated with the progression of atherosclerotic stiffness among Chinese women with diabetes, independent of a variety of confounding factors.

## 1. Introduction

Cardiovascular disease (CVD) is the leading cause of mortality worldwide. According to estimates from the 2013 Global Burden of Disease (GBD) Study, 17.3 million deaths were due to CVD in 2013 [1]. Compared to men, women have a higher prevalence of CVD [2]. However, the specific risk predictors for women of CVD are still not identified.

The well-established risk factors for CVD include obesity, diabetes, hypertension, metabolic syndrome (MetS), drinking, and smoking [3–5]. Some studies investigated an association between parity and CVD [6]. Ness et al. showed that the higher the number of pregnancies, the greater the incidence of CVD [7]. Similarly, Aggarwal et al. suggested that an increased number of childbirths were associated with left ventricular diastolic dysfunction [8]. Parity is also associated with diabetes and MetS components, such as high-density lipoprotein cholesterol (HDL-C) and fasting plasma glucose (FBG). Evidence from Tong-ji Dongfeng Cohort Study revealed that higher parity may increase the risk of diabetes, and Vladutiu et al. found a positive association between parity and selected components of MetS including HDL as well as FBG [9, 10]. During pregnancy, a variety of hormones changes significantly such as placental lactogen, progesterone, and cortisol. Additionally, significant alternations in physiology, including insulin resistance and fat accumulation, may induce the incidence of diabetes and MetS [11–13]. However, there are few studies about the association between childbirths and atherosclerosis known as a main cause of CVD in Chinese population, especially in population with diabetes or hypertension. To the best of our knowledge, only one study demonstrated a relationship between a history of delivery and atherosclerosis [14]. But the study only analyzed the difference between nulliparous women and parous women; it did not involve an in-depth study of the relationship between the number of childbirths and atherosclerosis, nor did it adequately adjust for confounding factors such as MetS components or conduct a stratified analysis to fully detect the interaction of diabetes or hypertension.

In view of this, compared with previous studies, our study not only investigated the relationship between the number of childbirths and the risk of progress of atherosclerosis after adequately adjusting for various risk factors, including history of pregnancy, hypertensive disorder complicating pregnancy (HDCP), gestational diabetes mellitus (GDM), occupation, education, marital status, and MetS components such as HDL, FBG, and low-density lipoprotein cholesterol (LDL-C) as well as triglycerides (TG) but also analyzed the association between diabetic and hypertensive subgroups. Some studies have reported that decreased pulse wave velocity (PWV), a risk factor for atherosclerosis, can reflect decreased arterial stiffness [15–17]. Because the change in PWV is easy, inexpensive, and noninvasive to measure, we selected it as an indicator of the progress of atherosclerosis stiffness in our community survey. Thus, the purpose of our study is to explore the association between the number of childbirths and the progress of atherosclerosis among Chinese women with hypertension or diabetes.

## 2. Methods

### 2.1. Study Population

All subjects who participated in a community longitudinal survey conducted in the communities of Shijingshan district, Beijing, China, were investigated. The present work was one part of the survey from the REACTION Study and lasted from November 2011 to August 2012 [18]. A total of 19,443 residents whose ages ranged from 38 to 88 years were recruited. The inclusion criteria included women who had gone through natural menopause and had experienced childbirth. The exclusion criteria included a history of ovariectomy, a history of uterus operations, a history of estrogen or sex hormone replacement therapy, a diagnosis of peripheral vascular disease, nulliparity, and some drugs such as statins and antiplatelet drugs. Women who did not provide exact numbers of childbirths or answer the questionnaire were excluded. After 3 years, we conducted follow-up visits to the subjects from April 2015 to October 2015. Informed consent was signed by all participants before the investigation began. This analysis included 1159 eligible subjects. The Chinese People's Liberation Army General Hospital Ethics Committee approved our study. This study adhered to the principles of the Declaration of Helsinki.

### 2.2. Data Collection

Data were collected by trained staff according to standard operational processes. The subjects were invited to complete a standard questionnaire that included basic information, level of education, dietary habits, occupation, lifestyle, and disease history. We calculated the participants' body mass index (BMI) using the equation BMI = weight (kg)/height (m)^2^.

We used an Omron HEM-7117 electronic sphygmomanometer with the standard calibration method and appropriate cuff sizes to measure systolic blood pressure (SBP) and diastolic blood pressure (DBP) after the subjects rested for five minutes. SBP and DBP were measured three times on the right arm, and the machine read the measurements for one minute or more. At the same time, the subjects' pulse was counted three times. Then, we calculated the mean SBP, mean DBP, and mean pulse for our analysis. The PWV of the subjects was measured after a 5-minute rest in the supine position. The trained staff used the Omron Colin BP-203RPEIII device to measure PWV automatically, each time on the left and right. The upper arm and ankle of the subject were wrapped with occlusion and monitoring cuffs matched to the scope sensor and the pulse waveforms of the bilateral arms and tibial arteries were simultaneously recorded to determine the initial increase in arm and tibial waveform (transit time, *Δ*Tba). According to subjects' height, the distance from the brachium to the ankle transmission was obtained. According to the following equation: Lb = 0.2195 × height of patients (cm) − 2.0734, the path length from the suprasternal notch to the brachium (Lb) was obtained. According to the following equation: La = (0.8129 × height of patients (cm) + 12.328), the path length from the suprasternal notch to the ankle (La) was obtained. The calculation of PWV value was based on the ratio of the transmission distance from the brachium to the ankle divided by the transit time: PWV = (La-Lb)/ΔTba. The higher PWV was used for analysis. The methodology has been described and validated [19].

We took the fasting samples of the subjects' blood and measured glucose, total cholesterol (TC), TG, and other biochemical indexes, such as low-density lipoprotein cholesterol (LDL-C), HDL-C, HbA_1c_, gamma-glutamyl transpeptidase (GGT), and creatinine concentrations. We performed the standard 75 g oral glucose tolerance test on all subjects. The professional staff centrifuged all blood samples for 30 minutes and stored them at −80°C. If the subjects had been diagnosed with diabetes, they were given a standard meal containing 80 g carbohydrates; if not, they received a standard 75 g glucose solution. Venous blood samples were collected to measure glucose 2 hours after the oral glucose tolerance test or the standard meal test. The abovementioned study variables were measured using a Hitachi Automatic Biochemical Analyzer. The risk factors for atherosclerosis, which have been reported previously, were defined as follows: hypertension: any self-reported history of hypertension or SBP ≥ 140 mmHg or DBP ≥ 90 mmHg; diabetes: FBG ≥ 7.0 mmol/L and 2 h postprandial plasma glucose (PBG) ≥ 11.1 mmol/L simultaneously or any self-reported history of diabetes mellitus; coronary heart diseases (CHD): any self-reported history of coronary heart disease; and hyperlipidemia: any self-reported history of hyperlipidemia.

Age at menopause, age at first gestation, pregnancy history, menopausal status, history of estrogen replacement therapy, and the number of childbirths were all collected with self-reported questionnaires. We excluded subjects with unclear or missing data for the number of childbirths and other variables.

According to the number of childbirths, the women were divided into three groups as follows: women with 1 birth, women with 2 births, and women with ≥3 births, respectively. To further analyze the relationship between other variables and arterial stiffness, the patients were classified into several subgroups based on history of hypertension and diabetes. According to history of hypertension and diabetes, the subjects were divided into two subgroups, respectively. The change in PWV was calculated as follows: (the change of PWV) = (PWV measured in 2015) − (PWV measured in 2011). The greater the PWV change, the more severe the atherosclerosis. A change of PWV > 0 indicates poor arterial compliance and atherosclerotic progression. No arterial stiffness progress was defined as a change of PWV ≤ 0, and progression of arterial stiffness was indicated by a change in PWV > 0. The outcome is the progression of arterial stiffness.

### 2.3. Statistical Analysis

In our study, we showed continuous variables of normal distribution as the mean ± SD and continuous of nonnormal distribution as median (25th percentile–75th percentile), and we showed categorical variables as percentages (%). We compared continuous variables of normal distribution using 1-way ANOVA and if continuous variables of nonnormal distribution using Kruskal-Wallis test. The Pearson chi-square test was applied to compare all categorical variables between the groups with different numbers of childbirths.

Logistical and linear regression analyses were applied to investigate the correlation between the number of childbirths and the progression of atherosclerotic stiffness. In this analysis, the group with a change in PWV ≤ 0 was regarded as the reference group. Adjusted model I was built to test the hypothesis that higher numbers of childbirths contributed to the progression of atherosclerosis in different ranges, independent of age, BMI, age at first gestation, age at menopause, TG, HDL-C, FBG, PBG, HbA_1c_, SBP, DBP, pulse, history of smoking, history of drinking, pregnancy history, history of CHD, history of hyperlipidemia, history of taking hypoglycemic drugs, and history of taking antihypertensive drugs. Based on the variables adjusted in adjusted model I, TC, marital status, level of education, occupation, and history of HDCP and GDM were added to the variables in adjusted model II. We also calculated the statistical power to confirm that the associations were robust.

To further explore the relationship between the number of childbirths and the risk of the progression of atherosclerotic stiffness, a stratification analysis was performed for history of diabetes and hypertension.

*P* value < 0.05 was considered statistically significant. We used Empower (R) (http://www.empowerstats.com, X & Y Solutions Inc., Boston, MA) and R (http://www.R-project.org) to analyze the data in our study.

## 3. Results

A total of 1159 postmenopausal women were included in our study. The characteristics of the subjects according to number of childbirths are shown in Table 1. A total of 527 women had given birth once, 383 women had given birth twice, and 249 women had given birth at least three times. The subjects who had given birth once had statistically significantly lower BMI, SBP, GGT, GFR, HbA_1c_, FBG, PBG, and PWV than the other two groups (Table 1).

**Figure 1:**
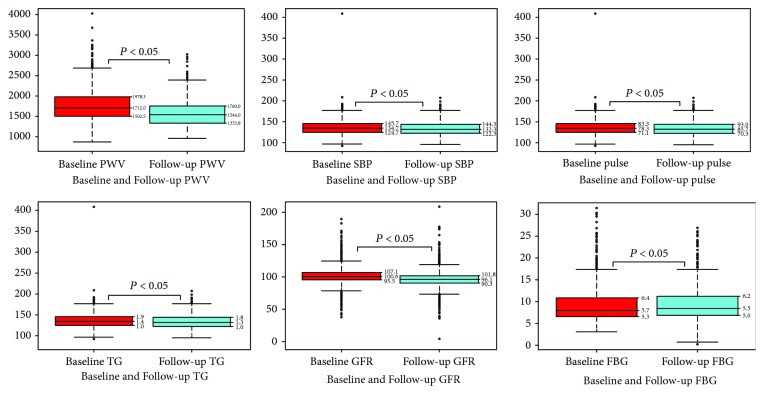
Box plots of baseline and follow-up PWV and the change variable along with medians and IQRs.

**Figure 2:**
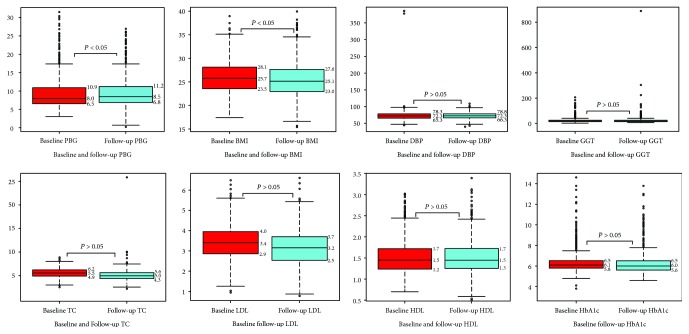
Box plots/histograms of baseline and follow-up PWV and the change variable along with means/medians and STD/IQRs.

**Figure 3:**
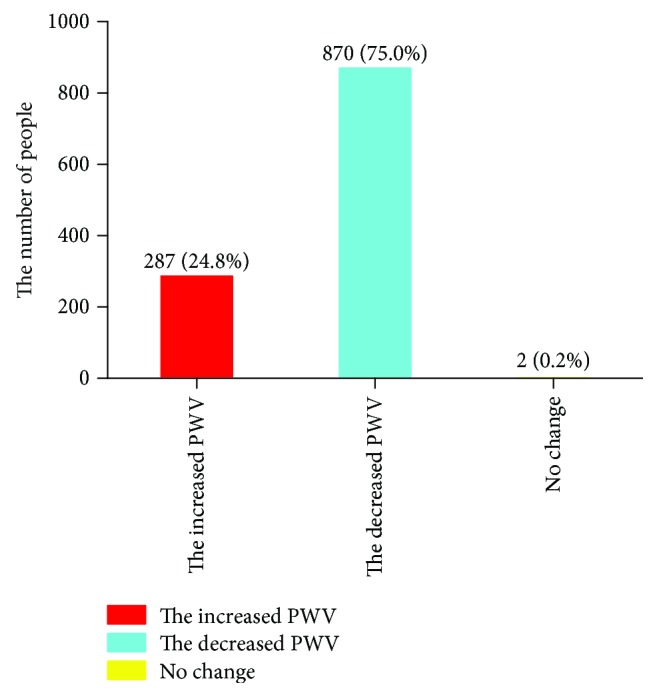
The number of people in the different PWV group.

Baseline and follow-up PWV as well as the change variable along with means/medians and STD/IQRs were shown in Figures 1 and 2. After 3-year follow-up, 287 (24.8%) women had increase in PWV, 870 (75.0%) women had decrease in PWV, and only 2 (0.2%) women had no change in Figure 3.

**Figure 4:**
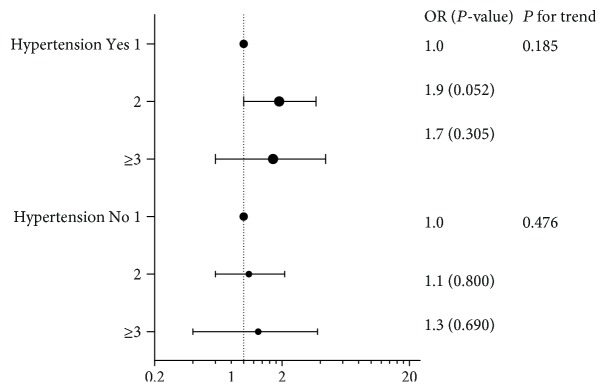
ORs for adjusted model II of association between number of births and the progress of atherosclerosis for SBP subgroups.

Figure 4 displays the results of logistic regressions for different subgroups of hypertension and linear regressions for hypertension were shown in Table S1.  Table S2 shows additional analyses analyzing follow-up PWV and including baseline PWV as a covariate. For different subgroups of hypertension, the relationship between the number of childbirths and the change in PWV was not statistically significant after adjusting for various confounding factors in logistic regressions or linear regressions.

**Figure 5:**
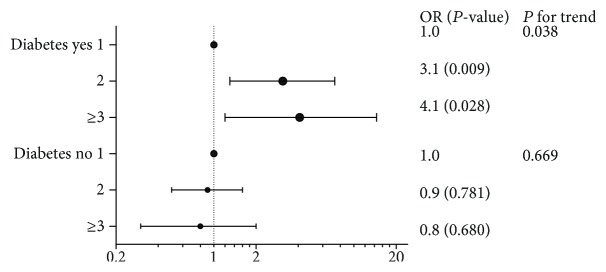
ORs for adjusted model II of association between number of births and the progress of atherosclerosis for diabetes subgroups.

Figure 5 and Table S3 display the results of the logistic regression models, including the nonadjusted model, which did not adjust for any variables, and adjusted model I, which adjusted for age, BMI, SBP, DBP, GGT, TG, LDL-C, HDL-C, FBG, PBG, HbA_1c_, CHD history, hyperlipidemia history, age at first gestation, age at menopause, number of pregnancies, smoking status, drinking status, antihypertensive drug use, and hypoglycemic drug use. In adjusted model I, among the subjects with diabetes, the OR of women with 2 childbirths was 3.5 (95% confidence interval 1.5, 7.9) compared with the women with 1 childbirth. Among the subjects with diabetes, the OR of women with ≥3 childbirths was 4.4 (95% confidence interval 1.3, 14.5) compared with women with 1 childbirth. In adjusted model II, among the subjects with diabetes, the OR of women with 2 childbirths was 3.1 (95% confidence interval 1.3, 7.2) compared with women with 1 childbirth. In addition, among the subjects with diabetes, the OR of women with ≥3 childbirths was 4.1 (95% confidence interval 1.2, 14.3) compared with women with 1 childbirth. In linear regression analysis, change in PWV as a continuous variable, the relationship was still significant (p for trend: 0.755 in nonadjusted model; 0.022 in model I; 0.038 in model II) among women with diabetes (Table S3). However, among the subjects without diabetes, the correlation was not present in logistic or linear regression analyses.  Table S4 displays the results of additional analyses analyzing follow-up PWV and including baseline PWV as a covariate, in which the correlation was not present in different subgroups of diabetes in Figure 5.

Sensitivity analyses were conducted, and when eGFR and breastfeeding time were added to logical regression models I and II, respectively, the OR basically did not change. The choice of confounding factors was based on the previously reported risk factors. In addition, covariate check and filters were introduced in the base model. The criteria for selecting covariates were as follows: when the covariate was introduced to the base model or eliminated from the complete model, the effect of the regression coefficients on X was greater than 10%.

The logistic and linear models presented and additional analysis analyzing follow-up PWV and including baseline PWV as a covariate were shown along with all covariates in supplemental material (Table S5–S16).

## 4. Discussion

Our findings are unique compared with previous studies. The novel findings of our study are that among women with diabetes, a higher number of births are significantly associated with a greater risk of progression of atherosclerosis stiffness, although for the whole group of participants, the relationship between the number of births and the risk of progression of atherosclerosis was not statistically significant. This result suggests that the relationship between the number of births and the progression of atherosclerosis was more significant among women with diabetes. Unlike the previous study, we adequately adjusted for confounding factors, including drinking status, HDCP, and GDM. To the best of our knowledge, this study is the first to explore the association between the number of childbirths and the risk of atherosclerotic stiffness progression in postmenopausal women with diabetes.

In the Framingham Heart Study, the number of pregnancies was associated with the risk of CVD. The more pregnancies, the higher the risk of CVD. After adjusting for the traditional risk factors, the relationship became weaker. Some previous studies reported similar findings that compared with nulliparous women who had never been pregnant, an increasing number of pregnancies was associated with a higher risk of CVD, especially in women with ≥6 pregnancies [7]. Similarly, in the NHEFS Study, although different methods were used to measure confounding factors, the conclusions were consistent. Aggarwal et al. showed that a higher number of childbirths were related to worse cardiac structure and dysfunction [8]. Beral revealed that a higher number of births were correlated with higher rates of uterine cancer, heart ischemia, and diabetes [20]. However, these studies only analyzed the differences in the incidence of CVD between nulliparous women and multiparous women. Without further analysis, in postmenopausal women with children, the association between the specific number of births and the risk of CVD is still unknown, especially in the population with hypertension or diabetes. To the best of our knowledge, only one study has analyzed the relationship between the number of childbirths and atherosclerotic stiffness, which is a main cause of CVD. It reported that among nonmenopausal women, parous women were more likely to have a slower progress of atherosclerotic stiffness than nulliparous women [14].

Our research and previous studies had many differences. First, our study was a cohort study that examined the change in PWV, which can reflect the progression of atherosclerosis in parous women during the follow-up period. The population of previous studies was premenopausal women, while our study targeted postmenopausal women. In addition, we conducted a stratified analysis based on the hypertension and diabetes history at baseline. The results showed that among women with diabetes, a higher number of childbirths were significantly associated with a greater risk of progression of atherosclerotic stiffness, independent of age; however, there was not a significant association among women with hypertension subgroups. When confounding factors were sufficiently adjusted, the relationship among women with diabetes still existed. The most significant points of our study are that the number of births is associated with progression of atherosclerotic stiffness in women with diabetes and that the association remains significant after adequately adjusting for HDCP, GDM, age at the first childbirth, and age at menopause, which were the confounding factors of CVD in some reported studies [20]. Additionally, our research takes sociological factors such as occupation, education, and marital status into account. These points have not been considered in previous studies. Furthermore, our research included a statistical power test to confirm the credibility of our results.

At present, the mechanism by which an increased number of births accelerates the progression of atherosclerotic stiffness is unclear. First, parity is likely to accelerate atherosclerotic progression through insulin resistance during pregnancy, and this alteration may last many years postpartum. Insulin resistance during pregnancy may contribute to a higher postpartum BMI, including abdominal obesity [21–23]. During gestation, abdominal obesity may lead to the accumulation of visceral adipose tissue (VAT) [24]. One study showed that during pregnancy, obese women may produce too many Th2 cytokines. Excessive production of inflammatory Th2 cytokines can contribute to endothelial vascular dysfunction [25]. These factors are likely to accelerate the formation and progression of atherosclerotic stiffness. Furthermore, studies have demonstrated that pregnant women have higher levels of TG, TC, and LDL-C, and these changes may result in increased oxidative stress, which is associated with atherosclerosis [26]. More births mean longer exposure to this environment, which may accelerate the formation and progression of atherosclerotic stiffness. The present study shows that in women with diabetes, higher numbers of births are significantly associated with a greater risk of progression of atherosclerotic stiffness. This may be due to the accumulation of VAT during pregnancy, which is reportedly related to the incidence of diabetes [27, 28]. However, the specific mechanism of this association is unclear and needs further exploration.

The strengths of the present study are that it was a cohort study, which reduced data bias as much as possible and it examined the impact of HDCP and GDM during pregnancy and pregnancy characteristics including pregnancy history and age at the first childbirth on the progression of atherosclerosis and also considered smoking and drinking status. It included a comprehensive consideration of sociological factors, such as occupation, education level, and marital status. In short, this is a comprehensive study. To the best of our knowledge, it is the first study to show the association between the number of births and the progression of atherosclerotic stiffness among Chinese community-based postmenopausal women with children.

In summary, the number of childbirths is associated with the progress of atherosclerotic stiffness. The more childbirths, the higher the risk of the progress of atherosclerotic stiffness in postmenopausal women with diabetes, independent of various confounding factors. Our research provides a new view of fertility. Given the reduction in the global fertility rate, is it right that every woman should have as many children as possible? When a female patient is seen at an outpatient clinic, reproductive history is often ignored, and most clinicians may pay more attention to biochemical indexes. It is significant for women with the more childbirths to control or supervise blood glucose as early as possible. For women with diabetes, the additional childbirths may increase the risk of the progression of atherosclerotic stiffness. Thus, early prevention and intervention in postpartum women may reduce the risk of the progression of atherosclerotic stiffness.

Our study has some limitations. Firstly, it was a cohort study with an inevitable amount of loss to follow-up. Due to the study design, there was no measurement of biochemical indexes during pregnancy. Because there is limited relevant literature and we did not design the study to examine the mechanisms underlying the age effect, this finding cannot be safely interpreted.

## Figures and Tables

**Table 1:** Characteristic of the subjects.

Variable	Total	Number of childbirths			*P* value
		1	2	≥3	
N	1159	527	383	249	
Age (years)	63.0 (59.0–69.0)	59.0 (58.0–61.0)	65.0 (62.0–69.0)	71.0 (69.0–74.0)	<0.001
BMI (kg/m^2^)	25.9 ± 3.5	25.4 ± 3.4	26.3 ± 3.4	26.2 ± 3.7	<0.001
SBP (mmHg)	134.0 (124.0–145.0)	131.0 (122.0–141.0)	136.0 (125.0–145.0)	141.0 (130.0–153.0)	<0.001
DBP (mmHg)	72.0 (65.0–78.0)	72.0 (66.0–78.0)	72.0 (65.0–78.0)	70.0 (63.0–77.0)	0.004
Pulse (bpm)	78.0 (71.0–85.0)	78.3 (71.7–85.0)	79.0 (71.0–85.0)	78.0 (72.0–85.0)	0.711
GGT (mmol/L)	18.2 (14.1–24.8)	17.9 (13.9–23.7)	18.5 (14.0–25.4)	18.3 (14.2–25.5)	0.499
TC (mmol/L)	5.6 ± 1.0	5.6 ± 0.9	5.6 ± 1.0	5.5 ± 1.1	0.660
TG (mmol/L)	1.4 (1.0-1.9)	1.4 (1.0-1.9)	1.4 (1.1–1.8)	1.4 (1.0-1.9)	0.866
GFR (mL/min/1.73m^2^)	100.6 (95.5–107.1)	100.4 (97.0–104.0)	101.0 (94.5–112.1)	101.6 (89.8–111.5)	0.227
LDL-C (mmol/L)	3.4 ± 0.9	3.4 ± 0.8	3.4 ± 0.9	3.4 ± 0.9	0.779
HDL-C (mmol/L)	1.5 ± 0.4	1.5 ± 0.4	1.5 ± 0.4	1.5 ± 0.4	0.701
FBG (mmol/L)	5.7 (5.3–6.4)	5.6 (5.3–6.2)	5.8 (5.3–6.5)	6.0 (5.4–7.1)	<0.001
PBG (mmol/L)	8.0 (6.5–10.9)	7.5 (6.4–9.8)	8.0 (6.5–10.9)	8.9 (7.0–12.6)	<0.001
HbA_1c_ (%)	6.1 (5.8–6.5)	6.0 (5.7–6.3)	6.1 (5.9–6.6)	6.2 (5.9–6.7)	<0.001
PWV (cm/s)	1712.0 (1502.5–1978.2)	1612.0 (1445.5–1826.5)	1766.0 (1548.0–2041.5)	1894.0 (1667.0–2190.0)	<0.001
Age at first gestation (years)	26.0 (24.0–28.0)	27.0 (26.0–29.0)	25.0 (23.0–27.0)	22.0 (21.0–25.0)	<0.001
Age at menopause (years)	50.0 (48.0–53.0)	50.0 (48.0–53.0)	50.0 (47.0–53.0)	50.0 (47.0–52.0)	0.015
Occupation, *n* (%)					**<0.001**
Worker	337 (29.0%)	101 (19.2%)	111 (28.9%)	125 (50.2%)	
Farmer	197 (17.0%)	34 (6.5%)	112 (29.2%)	51 (20.5%)	
Cadre	180 (16.0%)	180 (34.2%)	0 (0.0%)	0 (0.0%)	
Office and technical personnel, doctor, teacher	57 (4.9%)	55 (10.5%)	0 (0.0%)	2 (0.8%)	
Service worker	51 (4.4%)	51 (9.7%)	0 (0.0%)	0 (0.0%)	
Housewife	321 (27.7%)	104 (19.8%)	155 (40.4%)	62 (24.9%)	
Unemployed	10 (0.9%)	1 (0.1%)	3 (0.8%)	6 (2.4%)	
Other	6 (0.5%)	0 (0.0%)	3 (0.8%)	3 (1.2%)	
Education, *n* (%)					**<0.001**
Illiterate	50 (4.3%)	5 (0.9%)	9 (2.3%)	36 (14.6%)	
Primary school	162 (13.8%)	23 (4.4%)	56 (14.6%)	83 (33.6%)	
Junior high school	494 (42.2%)	263 (49.9%)	149 (38.9%)	80 (32.4%)	
Senior high school	279 (23.8%)	138 (26.2%)	109 (28.5%)	27 (10.9%)	
College	185 (15.8%)	98 (18.6%)	60 (15.7%)	21 (8.5%)	
Marriage status, *n* (%)					**<0.001**
Married	1027 (88.6%)	488 (92.6%)	341 (89.0%)	198 (79.5%)	
Widowed	118 (10.2%)	28 (5.3%)	40 (10.4%)	50 (20.1%)	
Separated	1 (0.1%)	1 (0.2%)	0 (0.0%)	0 (0.0%)	
Divorced	13 (1.1%)	10 (1.9%)	2 (0.5%)	1 (0.4%)	
Number of pregnancies, *n* (%)					**<0.001**
1	147 (12.7%)	147 (27.9%)	0 (0.0%)	0 (0.0%)	
2	343 (29.4%)	204 (38.7%)	138 (36.0%)	1 (0.4%)	
3	350 (29.9%)	136 (25.8%)	145 (37.9%)	69 (27.7%)	
4	214 (18.4%)	30 (5.7%)	81 (21.1%)	103 (41.4%)	
5	81 (6.9%)	6 (1.1%)	16 (4.2%)	59 (23.7%)	
6	21 (1.8%)	3 (0.6%)	2 (0.5%)	16 (6.4%)	
8	3 (0.3%)	1 (0.2%)	1 (0.3%)	1 (0.4%)	
HDCP, *n* (%)					**0.180**
Yes	86 (7.6%)	45 (8.5%)	29 (7.6%)	12 (4.8%)	
No	1043 (92.4%)	482 (91.5%)	354 (92.4%)	237 (95.2%)	
GDM, *n* (%)					**0.942**
Yes	4 (0.3%)	2 (0.4%)	1 (0.3%)	1 (0.4%)	
No	1155 (99.7%)	525 (99.6%)	382 (99.7%)	248 (99.6%)	
CHD					**0.003**
Yes	166 (14.3%)	56 (10.6%)	62 (16.2%)	48 (19.3%)	
No	993 (85.7%)	471 (89.4%)	321 (83.8%)	201 (80.7%)	
Hypertension, *n* (%)					**<0.001**
Yes	680 (58.7%)	265 (50.3%)	243 (63.4%)	172 (69.1%)	
No	479 (41.3%)	262 (49.7%)	140 (36.6%)	77 (30.9%)	
Diabetes					**<0.001**
Yes	434 (37.4%)	156 (29.6%)	155 (40.5%)	123 (49.4%)	
No	725 (62.6%)	371 (70.4%)	228 (59.5%)	126 (50.6%)	
Hyperlipidemia, *n* (%)					**0.962**
Yes	348 (30.0%)	159 (30.2%)	116 (30.3%)	73 (29.3%)	
No	811 (70.0%)	368 (69.8%)	267 (69.7%)	176 (70.7%)	
Antihypertensive drug use, *n* (%)					**0.006**
Yes	183 (15.8%)	65 (12.4%)	77 (20.2%)	41 (16.5%)	
No	973 (84.2%)	461 (87.6%)	304 (79.8%)	208 (83.5%)	
Hypoglycemic drug use, *n* (%)					**0.016**
Yes	154 (13.3%)	55 (10.4%)	55 (14.4%)	44 (17.7%)	
No	1005 (86.7%)	472 (89.6%)	328 (85.6%)	205 (82.3%)	
Drinking status, *n* (%)					**0.149**
No	1055 (91.0%)	471 (89.4%)	354 (92.4%)	230 (92.4%)	
Occasional drinkers	82 (7.1%)	48 (9.1%)	21 (5.5%)	13 (5.2%)	
Regular drinkers	22 (1.9%)	8 (1.5%)	8 (2.1%)	6 (2.4%)	
Smoking status, *n* (%)					**<0.001**
No	1121 (96.9%)	520 (99.0%)	375 (97.9%)	226 (90.8%)	
Occasional smokers	10 (0.9%)	1 (0.2%)	3 (0.8%)	6 (2.4%)	
Regular smokers	26 (2.2%)	4 (0.8%)	5 (1.3%)	17 (6.8%)	

The subject characteristic data are expressed as the mean ± SD for continuous variables and as percentages for categorical variables. BMI: body mass index; SBP: systolic blood pressure; DBP: diastolic blood pressure; GFR: glomerular filtration rate; TG: triglyceride; TC: total cholesterol; GGT: gamma-glutamyl transpeptidase; LDL-C: low-density lipoprotein cholesterol; HDL-C: high-density lipoprotein cholesterol; FBG: 0-hour fasting blood glucose; PBG: 2-hour postprandial blood glucose; PWV: pulse wave velocity.

## Additional Points

*Highlights*. The number of childbirths may be a predictor of long-term disease patterns in parous women. It is significant for women with more childbirths to control or supervise their blood glucose as early as possible. This is the first cohort study to show the association between the number of childbirths and the risk of progress of atherosclerosis in Chinese population with diabetes.

## Supplementary Materials

Supplementary Materials Table S1: logistic and linear regression analysis in hypertension history subgroup. Table S2: linear regression analysis between follow-up PWV and the number of childbirths in hypertension history subgroups. Table S3: logistic and linear regression analysis in diabetes history subgroups. Table S4: linear regression analysis between follow-up PWV and the number of childbirths in diabetes history subgroups. Table S5: linear regression analysis between follow-up PWV and the number of childbirths in hypertension history subgroups in model I. Table S6: linear regression analysis between follow-up PWV and the number of childbirths in hypertension history subgroups in model II. Table S7: logistical regression analysis between change in PWV as a category variable in hypertension subgroups in model II. Table S8: logistical regression analysis between change in PWV as a category variable in hypertension subgroups in model I. Table S9: linear regression analysis between change in PWV as a continuous variable in hypertension subgroups in model I. Table S10: linear regression analysis between change in PWV as a continuous variable in hypertension subgroups in model II. Table S11: linear regression analysis between follow-up PWV and the number of childbirths in diabetes history subgroups in model I. Table S12: linear regression analysis between follow-up PWV and the number of childbirths in diabetes history subgroups in model II. Table S13: logistical regression analysis between change in PWV as a category variable in diabetes subgroups in model I. Table S14: logistical regression analysis between change in PWV as a category variable in diabetes subgroups in model II. Table S15: linear regression analysis between change in PWV as a continuous variable in diabetes subgroups in model I. Table S16: linear regression analysis between change in PWV as a continuous variable in diabetes subgroups in model II.
