# Supplementary material for: Association between the Number of Childbirths and the Progress of Atherosclerosis among Women with Diabetes: A Cohort Study Based on Chinese Population
Source: Int J Endocrinol. 2019 Jan 27;2019:4874121. doi: 10.1155/2019/4874121 (PMC6369472; doi:10.1155/2019/4874121)
Supplement: Supplementary Materials — Table S1: logistic and linear regression analysis in hypertension history subgroup. Table S2: linear regression analysis between follow-up PWV and the number of childbirths in hypertension history subgroups. Table S3: logistic and linear regression analysis in diabetes history subgroups. Table S4: linear regression analysis between follow-up PWV and the number of childbirths in diabetes history subgroups. Table S5: linear regression analysis between follow-up PWV and the number of childbirths in hypertension history subgroups in model I. Table S6: linear regression analysis between follow-up PWV and the number of childbirths in hypertension history subgroups in model II. Table S7: logistical regression analysis between change in PWV as a category variable in hypertension subgroups in model II. Table S8: logistical regression analysis between change in PWV as a category variable in hypertension subgroups in model I. Table S9: linear regression analysis between change in PWV as a continuous variable in hypertension subgroups in model I. Table S10: linear regression analysis between change in PWV as a continuous variable in hypertension subgroups in model II. Table S11: linear regression analysis between follow-up PWV and the number of childbirths in diabetes history subgroups in model I. Table S12: linear regression analysis between follow-up PWV and the number of childbirths in diabetes history subgroups in model II. Table S13: logistical regression analysis between change in PWV as a category variable in diabetes subgroups in model I. Table S14: logistical regression analysis between change in PWV as a category variable in diabetes subgroups in model II. Table S15: linear regression analysis between change in PWV as a continuous variable in diabetes subgroups in model I. Table S16: linear regression analysis between change in PWV as a continuous variable in diabetes subgroups in model II. [file 4874121.f1.pdf]

## Supplemental materials

## Figure S1-S14

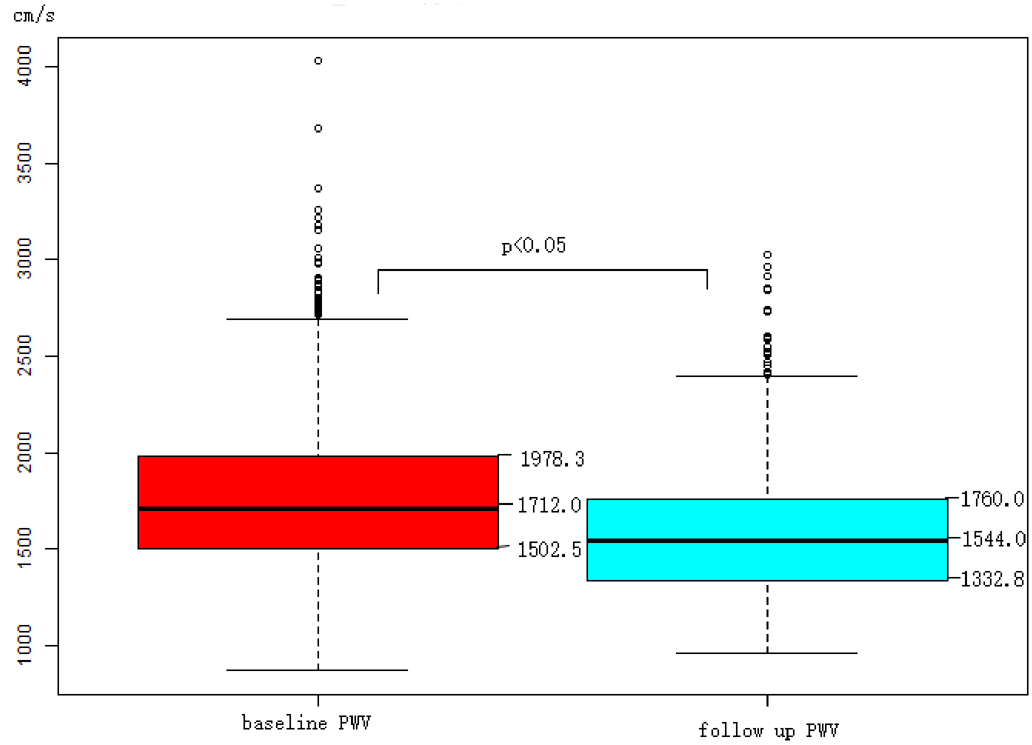


Figure S1.baseline and follow up PWV

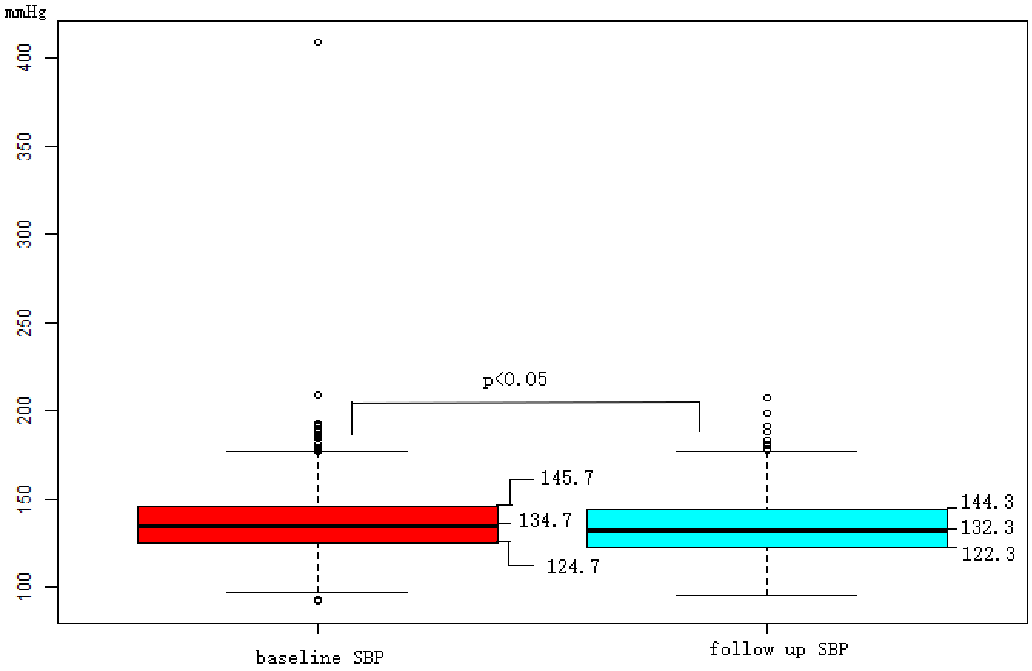


Figure S2. baseline and follow up SBP

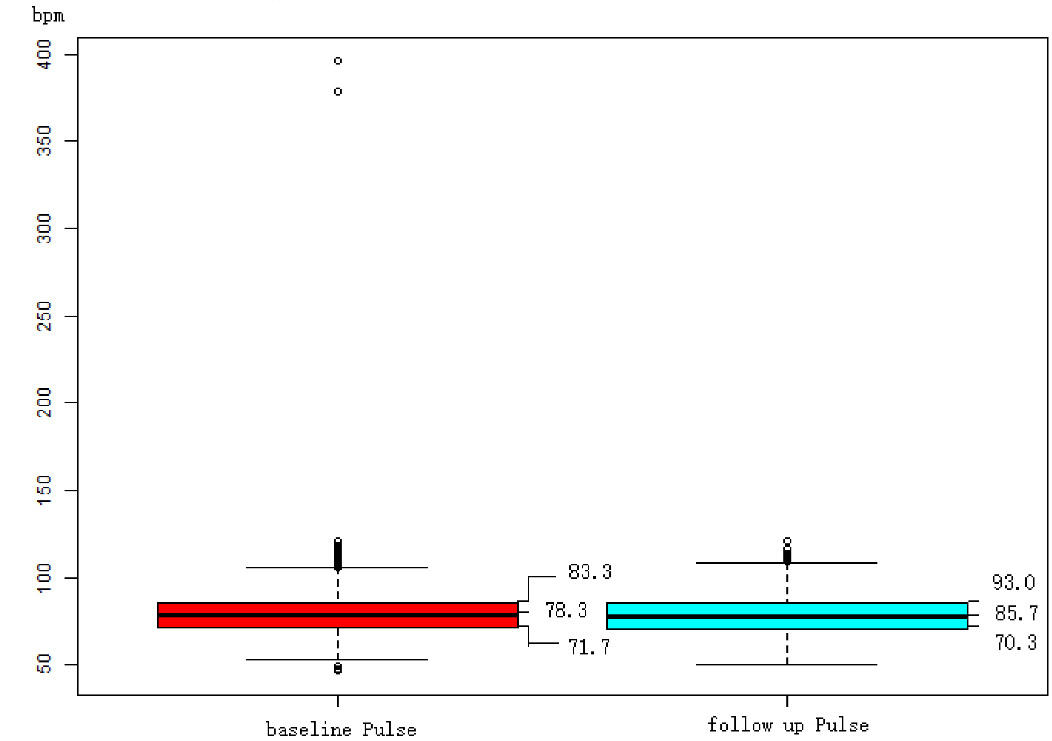

Fugire S3. baseline and follow up pulse

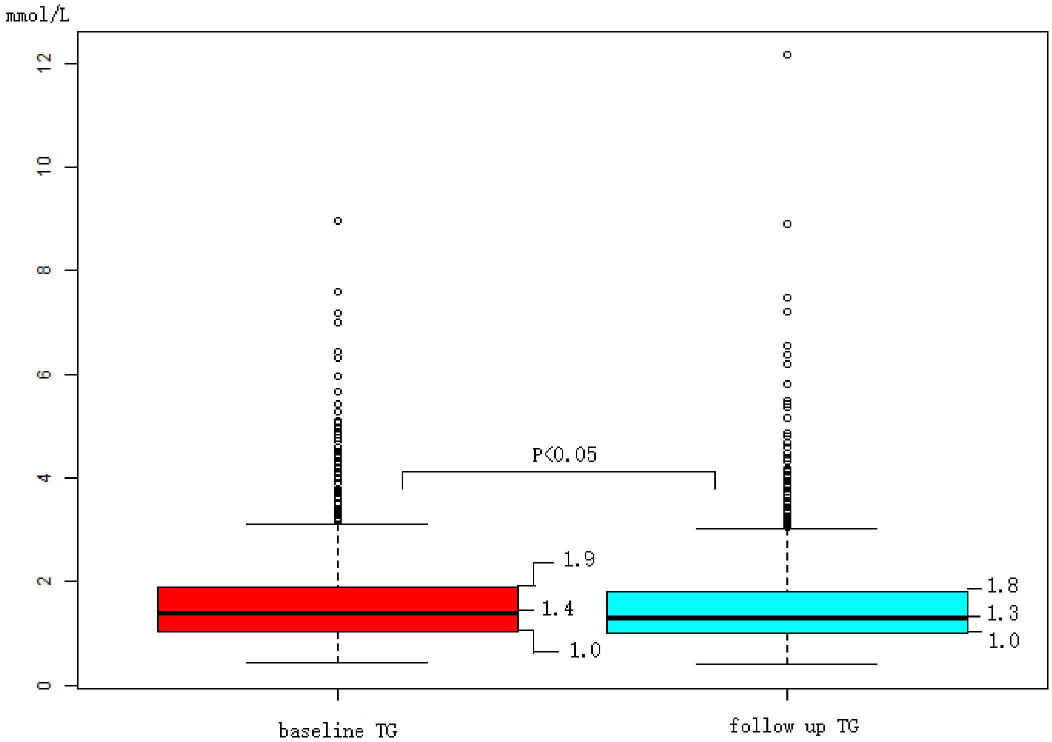

Figure S4.baseline and follow up TG

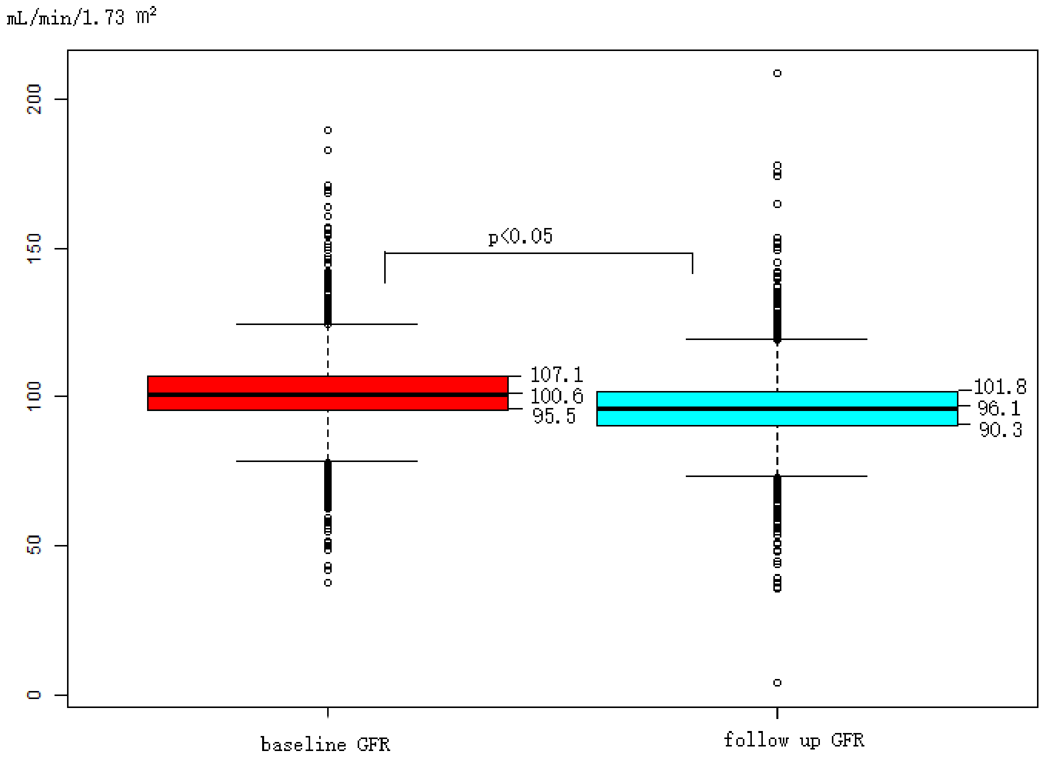

Figure S5.baseline and follow up GFR

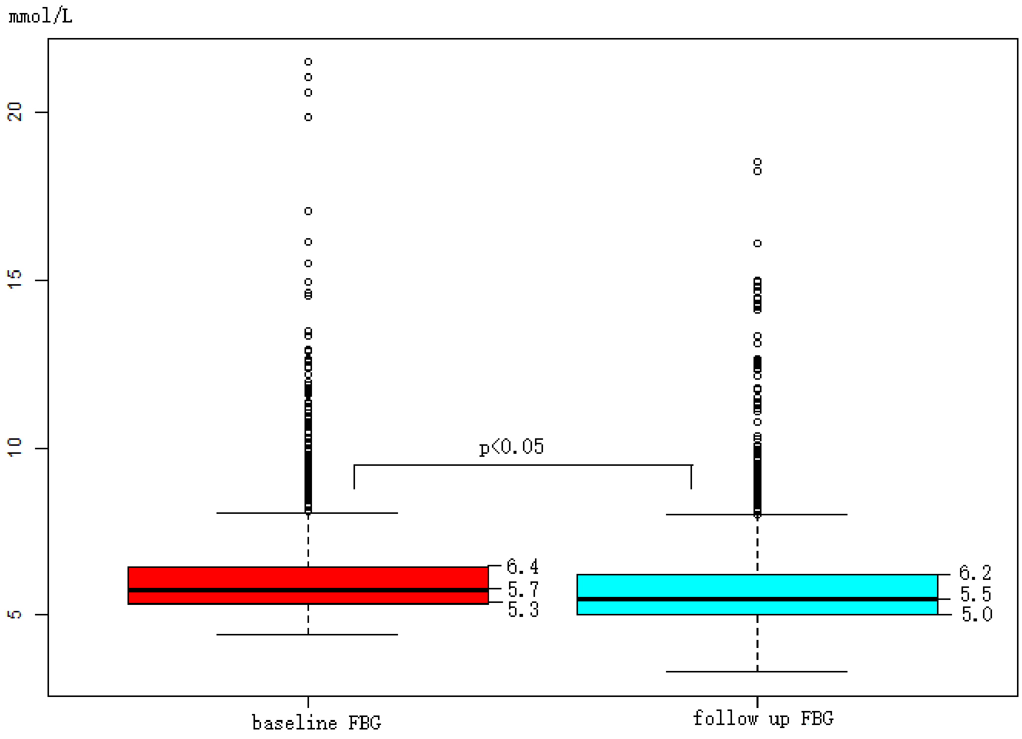

Figure S6.baseline and follow up FBG

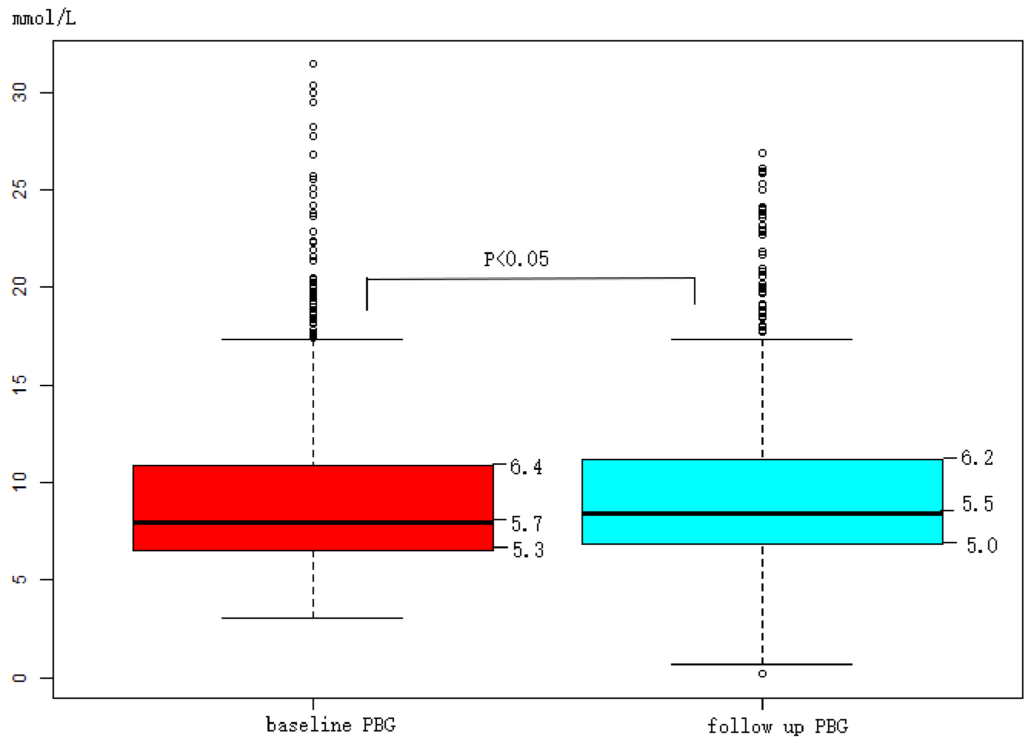


Figure S7.baseline and follow up PBG

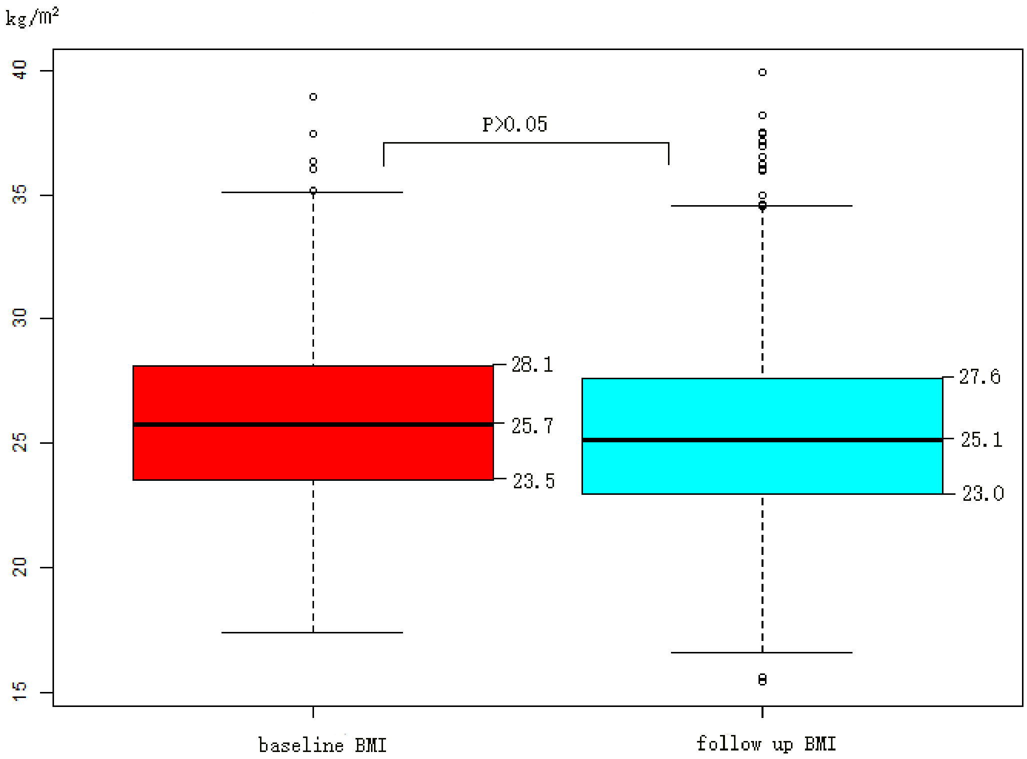


Figure S8.baseline and follow up BMI

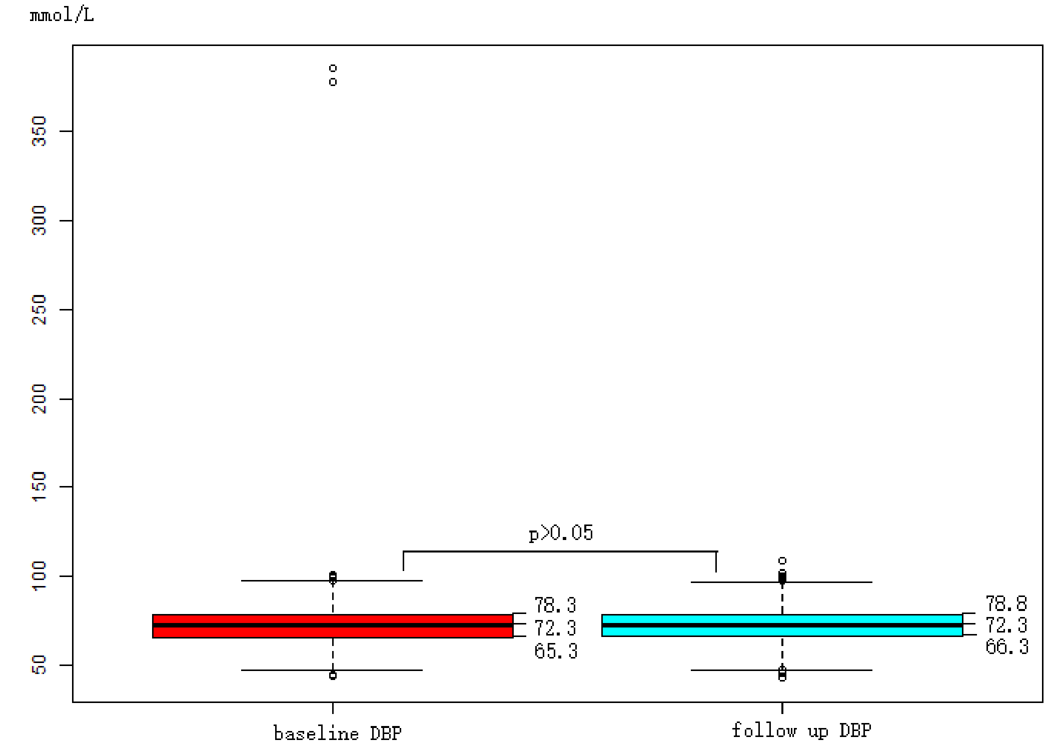

Figure S9.baseline and follow up DBP

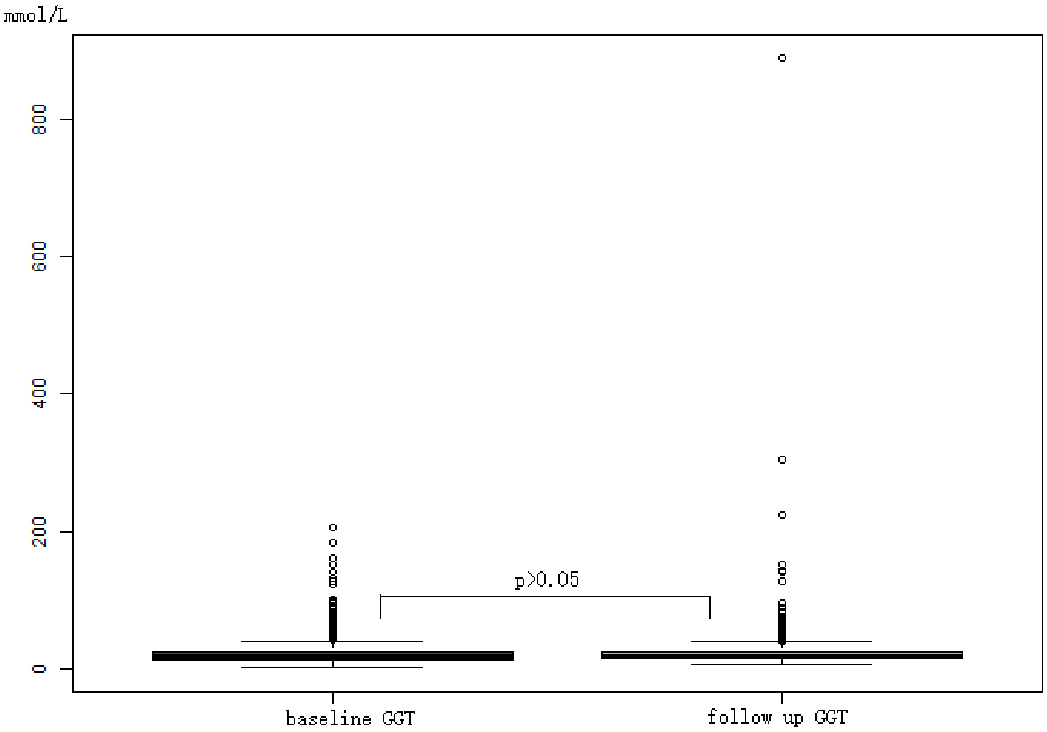

Figure S10. baseline and follow up GGT

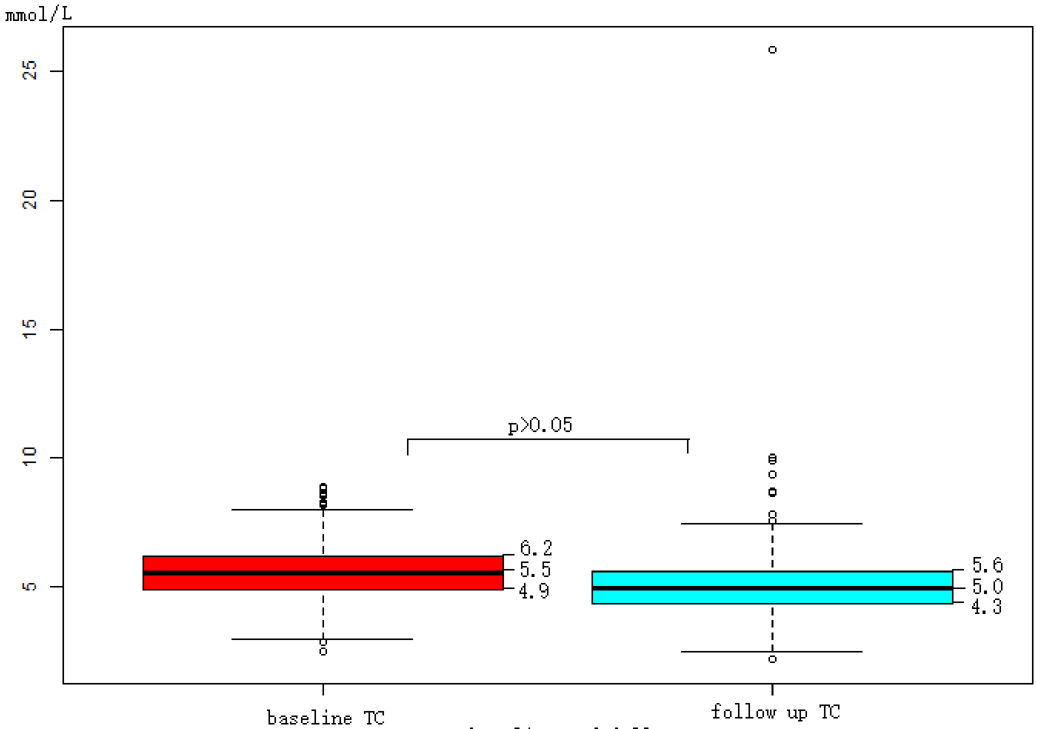

Figure S11.baseline and follow up TC

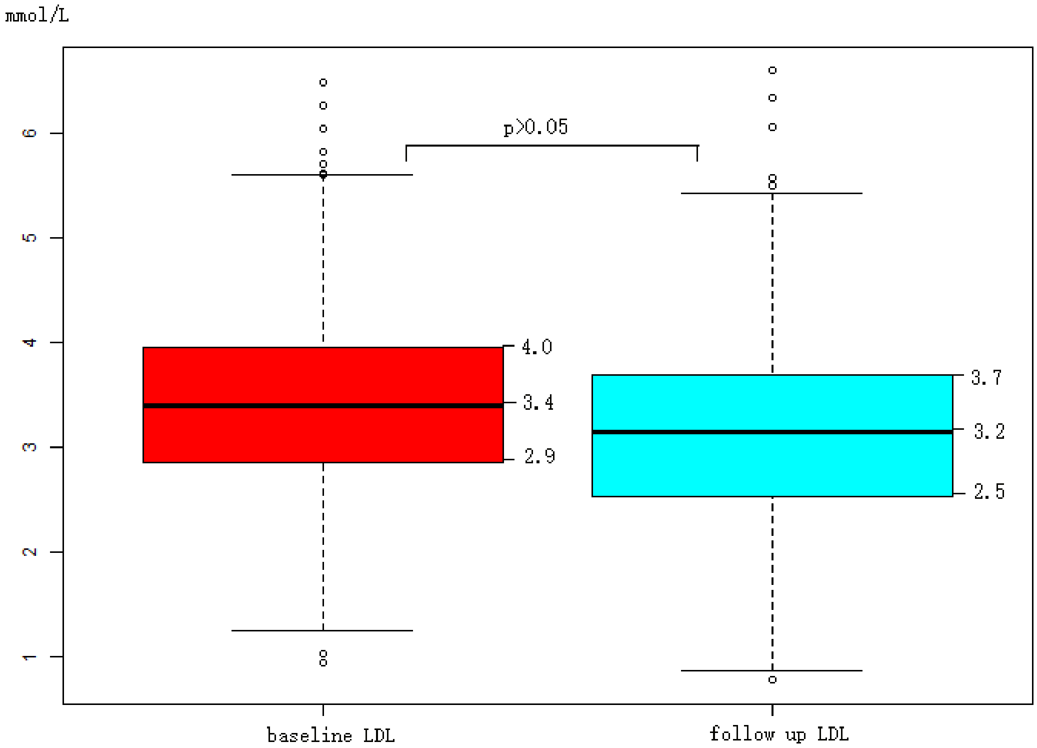

Figure S12.baseline and follow up LDL

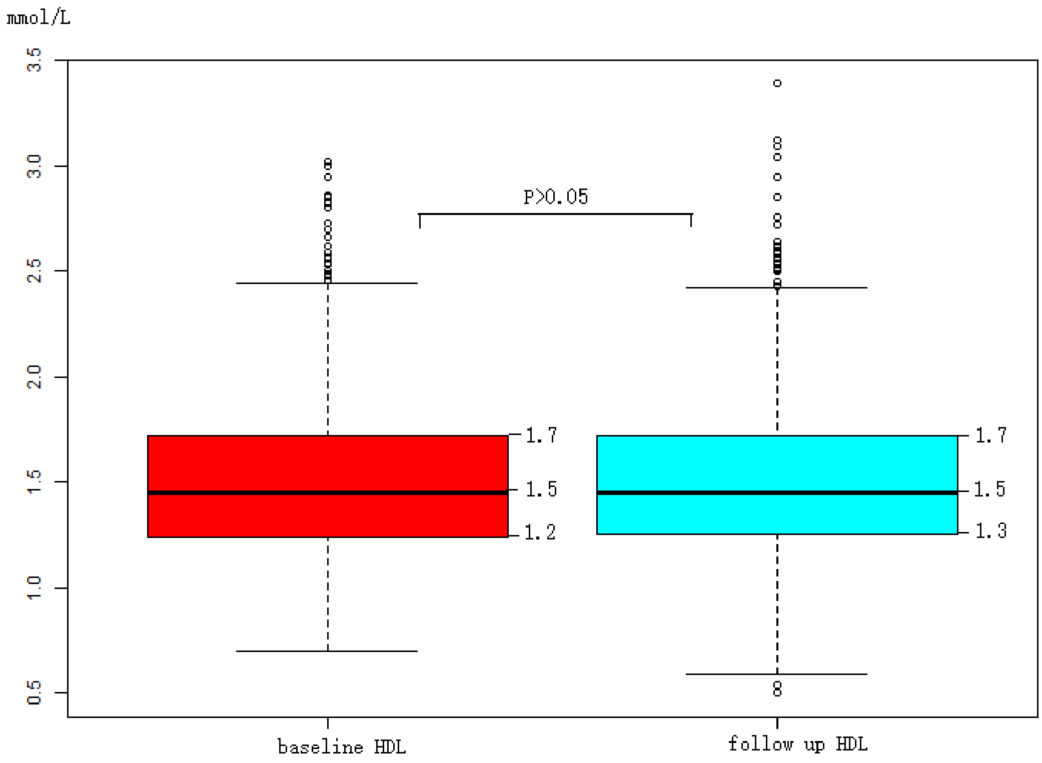


Figure S13.baseline and follow up HDL

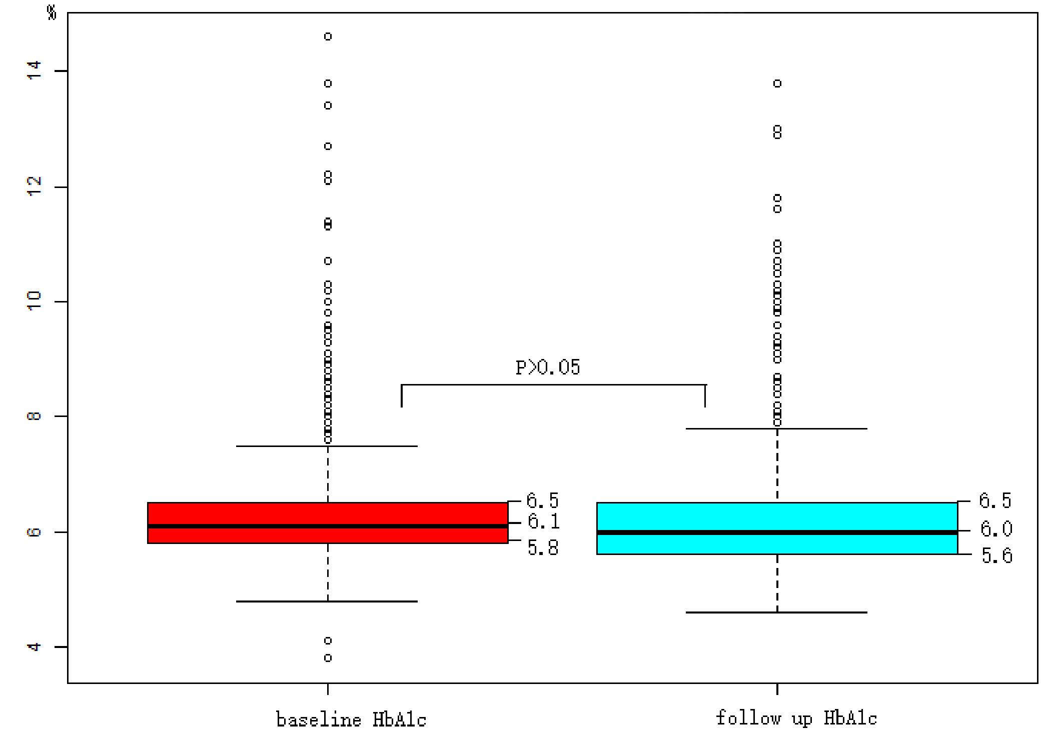


Figure S14.baseline and follow up HbA1c

## Table S1 Logistic and liner regression analysis in hypertension history subgroups

| Exposure | N | % | Non-adjusted | Adjust I | Adjust II |
|---|---|---|---|---|---|
| | | | OR (95%CI) *P*-value | OR (95%CI) *P*-value | OR (95%CI) *P*-value |
| Hypertension Yes | | | | | |
| PWV (cm/s) Category | | | | | |
| The number of childbirths | | | | | |
| 1 | 265 | 18.9 | 1.0 | 1.0 | 1.0 |
| 2 | 243 | 24.7 | 1.4 (0.9, 2.2) 0.112 | 1.8 (1.0, 3.5) 0.060 | 1.9 (1.0, 3.7) 0.052 |
| ≥3 | 172 | 25.6 | 1.5 (0.9, 2.3) 0.096 | 1.9 (0.7, 4.8) 0.197 | 1.7 (0.6, 4.4) 0.305 |
| P for trend | | | 0.066 | 0.229 | 0.309 |
| Continuous PWV (cm/s) | | | | | |
| The number of childbirths | | | | | |
| 1 | 265 | 18.9 | 0 | 0 | 0 |
| 2 | 243 | 24.7 | -9.6 (-71.3, 52.0) 0.759 | 71.2 (-15.2, 157.6) 0.107 | 77.0 (-11.3, 165.4) 0.088 |
| ≥3 | 172 | 25.6 | -23.2 (-91.2, 44.7) 0.503 | 102.8 (-26.0, 231.5) 0.118 | 96.2 (-36.6, 229.1) 0.156 |
| P for trend | | | 0.505 | 0.109 | 0.135 |
| Hypertension No | | | | | |
| PWV (cm/s) Category | | | | | |
| The number of childbirths | | | | | |
| 1 | 262 | 29.4 | 1.0 | 1.0 | 1.0 |
| 2 | 140 | 29.3 | 1.0 (0.6, 1.6) 0.983 | 1.1 (0.6, 2.2) 0.698 | 1.1 (0.6, 2.1) 0.800 |
| ≥3 | 77 | 26.0 | 0.8 (0.5, 1.5) 0.560 | 1.3 (0.4, 3.9) 0.613 | 1.3 (0.4, 3.8) 0.690 |

| | | | | | |
|---|---|---|---|---|---|
| **P for trend** | | | 0.492 | 0.612 | 0.729 |
| **Continuous PWV (cm/s)** | | | | | |
| **The number of childbirths** | | | | | |
| **1** | 262 | 29.4 | 0 | 0 | 0 |
| **2** | 140 | 29.3 | 7.0 (-47.3, 61.3) 0.801 | 36.1 (-37.6, 109.7) 0.337 | 35.8 (-39.4, 110.9) 0.352 |
| **≥3** | 77 | 26.0 | -47.6 (-114.8, 19.6) 0.165 | 47.5 (-73.4, 168.4) 0.441 | 46.8 (-76.3, 169.8) 0.457 |
| **P for trend** | | | 0.277 | 0.364 | 0.380 |
| **Total** | | | | | |
| **PWV (cm/s) Category** | | | | | |
| **The number of childbirths** | | | | | |
| **1** | 527 | 24.1 | 1.0 | 1.0 | 1.0 |
| **2** | 383 | 26.4 | 1.2 (0.9, 1.6) 0.282 | 1.4 (0.9, 2.2) 0.107 | 1.4 (0.9, 2.2) 0.124 |
| **≥3** | 249 | 25.7 | 1.2 (0.8, 1.7) 0.395 | 1.6 (0.8, 3.1) 0.189 | 1.6 (0.8, 3.1) 0.195 |
| **P for trend** | | | 0.346 | 0.179 | 0.177 |
| **Continuous PWV (cm/s)** | | | | | |
| **The number of childbirths** | | | | | |
| **1** | 527 | 24.1 | 0 | 0 | 0 |
| **2** | 383 | 26.4 | -3.5 (-45.9, 38.9) 0.872 | 52.5 (-5.3, 110.3) 0.076 | 55.0 (-3.9, 113.8) 0.067 |
| **≥3** | 249 | 25.7 | -30.7 (-79.4, 18.1) 0.218 | 81.7 (-7.0, 170.5) 0.071 | 83.5 (-7.1, 174.1) 0.071 |
| **P for trend** | | | 0.256 | 0.058 | 0.056 |

Non-adjusted model adjusted for nothing

Adjusted model I adjusted for age; BMI; SBP; DBP; TG; GGT; LDL-C; HDL-C; FBG; PBG; HbA1c ; CHD history; hyperlipidemia history; age at first gestation; age at menopause; number of pregnancies; smoking status; drinking status; antihypertensive drug use; hypoglycemic drug use

Adjusted model II adjusted for adjusted model I + TC; hypertensive disorder complicating pregnancy (HDCP); gestational diabetes mellitus (GDM)

## Table S2. Liner regression analysis between follow up PWV and the number of childbirths in hypertension history subgroups

| Exposure | N | % | Non-adjusted | Adjust I | Adjust II |
|---|---|---|---|---|---|
| | | | OR (95%CI) *P*-value | OR (95%CI) *P*-value | OR (95%CI) *P*-value |
| **Hypertension Yes** | | | | | |
| **The number of childbirths** | | | | | |
| **1** | 265 | 18.9 | 0 | 0 | 0 |
| **2** | 243 | 24.7 | 127.7 (73.6, 181.9) <0.001 | 55.3 (-12.0, 122.7) 0.108 | 54.1 (-14.4, 122.6) 0.122 |
| **≥3** | 172 | 25.6 | 205.8 (146.1, 265.5) <0.001 | 74.4 (-26.0, 174.7) 0.147 | 66.0 (-37.0, 169.0) 0.210 |
| **P for trend** | | | <0.001 | 0.134 | 0.185 |
| **Hypertension No** | | | | | |
| **The number of childbirths** | | | | | |
| **1** | 262 | 29.4 | 0 | 0 | 0 |
| **2** | 140 | 29.3 | 96.2 (37.7, 154.6) 0.001 | 37.7 (-27.9, 103.4) 0.261 | 35.2 (-32.2, 102.5) 0.307 |
| **≥3** | 77 | 26.0 | 212.9 (140.5, 285.2) <0.001 | 35.8 (-72.0, 143.7) 0.515 | 27.6 (-82.6, 137.9) 0.623 |
| **P for trend** | | | <0.001 | 0.383 | 0.476 |
| **Total** | | | | | |
| **Continuous PWV (cm/s)** | | | | | |

| | | | | | |
|---|---|---|---|---|---|
| **The number of childbirths** | | | | | |
| **1** | 527 | 24.1 | 0 | 0 | 0 |
| **2** | 383 | 26.4 | 115.0 (75.2, 154.8) <0.001 | 47.2 (0.0, 94.5) 0.050 | 45.4 (-2.5, 93.4) 0.064 |
| **≥3** | 249 | 25.7 | 206.3 (160.5, 252.1) <0.001 | 59.8 (-12.7, 132.3) 0.106 | 56.3 (-17.6, 130.2) 0.136 |
| **P for trend** | | | <0.001 | 0.078 | 0.100 |

Non-adjusted model adjusted for nothing

Adjusted model I adjusted for age; BMI; SBP; DBP; TG; GGT; LDL-C; HDL-C; FBG; PBG; HbA1c ; baseline PWV; CHD history; hyperlipidemia history; age at first gestation; age at menopause; number of pregnancies; smoking status; drinking status; antihypertensive drug use; hypoglycemic drug use

Adjusted model II adjusted for adjusted model I + TC; hypertensive disorder complicating pregnancy (HDCP); gestational diabetes mellitus (GDM)

## Table S3. Logistic and liner regression analysis in diabetes history subgroups

| **Exposure** | **N** | **%** | **Non-adjusted** | **Adjust I** | **Adjust II** |
|---|---|---|---|---|---|
| | | | **OR (95%CI) *P*-value** | **OR (95%CI) *P*-value** | **OR (95%CI) *P*-value** |
| **Diabetes Yes** | | | | | |
| **PWV (cm/s) Category** | | | | | |
| **The number of childbirths** | | | | | |
| **1** | 156 | 17.3 | 1.0 | 1.0 | 1.0 |
| **2** | 155 | 29.7 | 2.0 (1.2, 3.5) 0.011 | 3.5 (1.5, 7.9) 0.003 | 3.1 (1.3, 7.2) 0.009 |
| **≥3** | 123 | 26.8 | 1.8 (1.0, 3.1) 0.056 | 4.4 (1.3, 14.5) 0.015 | 4.1 (1.2, 14.3) 0.028 |
| **P for trend** | | | 0.050 | 0.032 | 0.017 |
| **Continuous PWV (cm/s)** | | | | | |
| **The number of childbirths** | | | | | |

| | | | | | |
|---|---|---|---|---|---|
| **1** | 156 | 17.3 | 0 | 0 | 0 |
| **2** | 155 | 29.7 | 58.2 (-23.3, 139.6) 0.162 | 130.3 (22.4, 238.1) 0.018 | 105.1 (-6.1, 216.3) 0.065 |
| **≥3** | 123 | 26.8 | -19.6 (-106.1, 67.0) 0.658 | 140.5 (-21.0, 302.1) 0.089 | 118.1 (-48.5, 284.7) 0.166 |
| **P for trend** | | | 0.755 | 0.022 | 0.038 |
| **Diabetes No** | | | | | |
| **PWV (cm/s) Category** | | | | | |
| **The number of childbirths** | | | | | |
| **1** | 371 | 27.0 | 1.0 | 1.0 | 1.0 |
| **2** | 228 | 24.1 | 0.9 (0.6, 1.3) 0.443 | 1.0 (0.6, 1.7) 0.910 | 0.9 (0.5, 1.6) 0.781 |
| **≥3** | 126 | 24.6 | 0.9 (0.6, 1.4) 0.605 | 1.0 (0.4, 2.4) 0.925 | 0.8 (0.3, 2.1) 0.680 |
| **P for trend** | | | 0.442 | 0.997 | 0.669 |
| **Continuous PWV (cm/s)** | | | | | |
| **The number of childbirths** | | | | | |
| **1** | 371 | 27.0 | 0 | 0 | 0 |
| **2** | 228 | 24.1 | -54.1 (-102.9, -5.4) 0.030 | 10.1 (-58.7, 78.9) 0.773 | 11.1 (-58.9, 81.2) 0.755 |
| **≥3** | 126 | 24.6 | -48.0 (-107.7, 11.8) 0.116 | 59.1 (-49.4, 167.5) 0.286 | 45.2 (-65.8, 156.2) 0.425 |
| **P for trend** | | | 0.042 | 0.496 | 0.562 |
| **Total** | | | | | |
| **PWV (cm/s) Category** | | | | | |
| **The number of childbirths** | | | | | |
| **1** | 527 | 24.1 | 1.0 | 1.0 | 1.0 |
| **2** | 383 | 26.4 | 1.1 (0.8, 1.5) 0.404 | 1.4 (0.9, 2.2) 0.122 | 1.4 (0.9, 2.1) 0.182 |

| | | | | | |
|---|---|---|---|---|---|
| **≥3** | 249 | 25.7 | 1.1 (0.8, 1.6) 0.570 | 1.6 (0.8, 3.1) 0.198 | 1.5 (0.8, 3.0) 0.239 |
| **P for trend** | | | 0.529 | 0.168 | 0.116 |
| **Continuous PWV (cm/s)** | | | | | |
| **The number of childbirths** | | | | | |
| **1** | 527 | 24.1 | 0 | 0 | 0 |
| **2** | 383 | 26.4 | -13.7 (-56.6, 29.2) 0.532 | 52.8 (-5.0, 110.6) 0.074 | 50.3 (-8.2, 108.8) 0.092 |
| **≥3** | 249 | 25.7 | -43.0 (-92.5, 6.5) 0.089 | 87.2 (-1.5, 175.9) 0.054 | 77.7 (-12.7, 168.1) 0.092 |
| **P for trend** | | | 0.097 | 0.023 | 0.029 |

Non-adjusted model adjusted for nothing

Adjusted model I adjusted for age; BMI; SBP; DBP; TG; GGT; LDL-C; HDL-C; FBG; PBG; HbA1c ; CHD history; hyperlipidemia history; age at first gestation; age at menopause; number of pregnancies; smoking status; drinking status; antihypertensive drug use; hypoglycemic drug use

Adjusted model II adjusted for adjusted model I + TC; hypertensive disorder complicating pregnancy (HDCP); gestational diabetes mellitus (GDM)

## Table S4. Liner regression analysis between follow up PWV and the number of childbirths in diabetes history subgroups

| Exposure | N | % | Non-adjusted | Adjust I | Adjust II |
|---|---|---|---|---|---|
| | | | OR (95%CI) *P*-value | OR (95%CI) *P*-value | OR (95%CI) *P*-value |
| Diabetes Yes | | | | | |
| The number of childbirths | | | | | |
| 1 | 156 | 17.3 | 0 | 0 | 0 |
| 2 | 155 | 29.7 | 115.4 (45.9, 184.8) 0.001 | 50.9 (-29.5, 131.4) 0.216 | 46.0 (-37.3, 129.3) 0.280 |
| ≥3 | 123 | 26.8 | 189.6 (115.7, 263.4) <0.001 | 40.5 (-79.6, 160.6) 0.509 | 36.2 (-88.5, 160.9) 0.569 |
| P for trend | | | <0.001 | 0.440 | 0.501 |
| Diabetes No | | | | | |
| The number of childbirths | | | | | |
| 1 | 371 | 27.0 | 0 | 0 | 0 |
| 2 | 228 | 24.1 | 133.0 (82.9, 183.1) <0.001 | 36.7 (-22.6, 96.0) 0.225 | 33.0 (-27.4, 93.3) 0.285 |
| ≥3 | 126 | 24.6 | 247.5 (186.0, 308.9) <0.001 | 61.9 (-31.8, 155.7) 0.196 | 47.1 (-48.4, 142.6) 0.334 |
| P for trend | | | <0.001 | 0.175 | 0.293 |
| Total | | | | | |
| Continuous PWV (cm/s) | | | | | |
| The number of childbirths | | | | | |
| 1 | 527 | 24.1 | 0 | 0 | 0 |

| | | | | | |
|---|---|---|---|---|---|
| **2** | 383 | 26.4 | 128.0 (87.4, 168.6) <0.001 | 47.3 (0.2, 94.4) 0.049 | 46.3 (-1.6, 94.2) 0.058 |
| **≥3** | 249 | 25.7 | 222.8 (175.9, 269.6) <0.001 | 62.0 (-10.5, 134.5) 0.094 | 58.5 (-15.4, 132.5) 0.121 |
| **P for trend** | | | <0.001 | 0.069 | 0.088 |

Non-adjusted model adjusted for nothing

Adjusted model I adjusted for age; BMI; SBP; DBP; TG; GGT; LDL-C; HDL-C; FBG; PBG; HbA1c ; baseline PWV; CHD history; hyperlipidemia history; age at first gestation; age at menopause; number of pregnancies; smoking status; drinking status; antihypertensive drug use; hypoglycemic drug use

Adjusted model II adjusted for adjusted model I + TC; hypertensive disorder complicating pregnancy (HDCP); gestational diabetes mellitus (GDM)

## Table S5. Liner regression analysis between follow up PWV and the number of childbirths in hypertension history subgroups in Model I

| Exposure | Hypertension Yes<br>B (95%CI) *P*-value | Hypertension No<br>B (95%CI) *P*-value | Total<br>B (95%CI) *P*-value |
|---|---|---|---|
| **Age** | 4.4 (-1.6, 10.3) 0.150 | 7.8 (0.5, 15.0) 0.037 | 5.4 (0.9, 9.9) 0.019 |
| **BMI** | -6.8 (-13.4, -0.2) 0.045 | -5.2 (-13.1, 2.8) 0.205 | -5.5 (-10.5, -0.5) 0.031 |
| **SBP** | 0.2 (-1.3, 1.7) 0.815 | 0.4 (-2.3, 3.2) 0.771 | 0.2 (-1.1, 1.5) 0.741 |
| **DBP** | 0.0 (-1.3, 1.4) 0.985 | 0.7 (-2.7, 4.1) 0.671 | 0.0 (-1.2, 1.2) 0.951 |
| **TG** | 16.4 (-12.2, 45.0) 0.260 | 3.2 (-28.0, 34.5) 0.839 | 11.4 (-9.5, 32.3) 0.286 |
| **GGT** | -0.9 (-2.1, 0.3) 0.128 | 0.3 (-1.2, 1.7) 0.723 | -0.5 (-1.4, 0.4) 0.266 |
| **LDL_C** | -13.2 (-38.4, 12.1) 0.306 | -9.4 (-37.2, 18.4) 0.508 | -9.0 (-27.7, 9.7) 0.344 |
| **HDL_C** | -15.4 (-85.6, 54.9) 0.668 | 9.4 (-64.3, 83.1) 0.804 | -10.0 (-60.5, 40.4) 0.697 |
| **FBG** | -0.8 (-23.9, 22.4) 0.949 | -10.9 (-34.5, 12.7) 0.366 | -7.7 (-23.9, 8.4) 0.350 |
| **PBG** | 3.5 (-4.2, 11.3) 0.372 | 9.0 (-1.1, 19.0) 0.081 | 5.9 (-0.2, 11.9) 0.057 |
| **HbA1c** | -5.1 (-45.1, 35.0) 0.805 | 13.3 (-27.7, 54.3) 0.526 | 0.1 (-27.9, 28.0) 0.996 |
| **Baseline PWV** | 0.4 (0.3, 0.4) <0.001 | 0.5 (0.4, 0.6) <0.001 | 0.4 (0.4, 0.5) <0.001 |
| **The number of childbirths** | | | |
| **1** | 0 | 0 | 0 |
| **2** | 55.3 (-12.0, 122.7) 0.108 | 37.7 (-27.9, 103.4) 0.261 | 47.2 (0.0, 94.5) 0.050 |
| **3** | 74.4 (-26.0, 174.7) 0.147 | 35.8 (-72.0, 143.7) 0.515 | 59.8 (-12.7, 132.3) 0.106 |
| **CHD history** | | | |
| **Yes** | 0 | 0 | 0 |
| **No** | -32.0 (-88.5, 24.5) 0.268 | -96.1 (-184.0, -8.1) 0.033 | -46.7 (-92.7, -0.6) 0.047 |
| **hyperlipidemia history** | | | |
| **Yes** | 0 | 0 | 0 |

| | | | |
|---|---|---|---|
| **No** | 28.2 (-19.4, 75.9) 0.246 | -22.2 (-75.1, 30.7) 0.411 | 3.8 (-31.2, 38.7) 0.833 |
| **age at first gestation** | -0.3 (-6.2, 5.6) 0.924 | 2.1 (-5.0, 9.1) 0.565 | -0.1 (-4.7, 4.4) 0.950 |
| **age at menopause** | 0.5 (-1.9, 2.9) 0.683 | -0.6 (-2.9, 1.8) 0.628 | 0.0 (-1.7, 1.7) 0.991 |
| **number of pregnancies** | | | |
| **0** | 0 | 0 | 0 |
| **1** | 0 | 0 | 0 |
| **2** | 18.0 (-60.8, 96.7) 0.655 | -23.6 (-93.5, 46.2) 0.507 | 0.3 (-52.6, 53.2) 0.991 |
| **3** | -1.7 (-84.0, 80.5) 0.967 | -33.6 (-108.5, 41.3) 0.380 | -17.6 (-73.2, 38.0) 0.536 |
| **4** | 4.1 (-90.4, 98.6) 0.932 | -87.3 (-177.8, 3.3) 0.060 | -32.2 (-97.7, 33.2) 0.334 |
| **5** | 14.2 (-98.4, 126.9) 0.804 | -1.7 (-146.6, 143.3) 0.982 | 9.0 (-76.5, 94.4) 0.837 |
| **6** | 2.8 (-188.6, 194.2) 0.977 | 102.6 (-147.7, 353.0) 0.422 | 11.6 (-136.1, 159.2) 0.878 |
| **8** | -320.4 (-634.3, -6.5) 0.046 | 0 | -333.4 (-626.8, -40.0) 0.026 |
| **smoking status** | | | |
| **No** | 0 | 0 | 0 |
| **Occasional smokers** | -106.8 (-328.1, 114.5) 0.345 | 114.5 (-97.6, 326.5) 0.291 | 5.6 (-146.6, 157.9) 0.942 |
| **Regular smokers** | -97.5 (-295.2, 100.2) 0.334 | 5.4 (-121.9, 132.8) 0.933 | -23.2 (-132.3, 85.8) 0.677 |
| **drinking status** | | | |
| **No** | 0 | 0 | 0 |
| **Occasional drinkers** | 23.3 (-69.9, 116.5) 0.625 | -70.3 (-145.8, 5.3) 0.069 | -26.3 (-85.8, 33.1) 0.385 |
| **Regular drinkers** | -5.9 (-169.0, 157.2) 0.943 | 114.9 (-41.1, 270.9) 0.150 | 33.0 (-79.1, 145.0) 0.564 |
| **hypoglycemic drug** | | | |
| **Yes** | 0 | 0 | 0 |
| **No** | -13.6 (-79.6, 52.4) 0.686 | 79.9 (-23.1, 182.9) 0.129 | -1.9 (-56.0, 52.1) 0.944 |

Adjusted model I adjusted for age; BMI; SBP; DBP; TG; GGT; LDL-C; HDL-C; FBG; PBG; HbA1c; baseline PWV; CHD history; hyperlipidemia history; age at first gestation; age at menopause; number of pregnancies; smoking status; drinking status; antihypertensive drug use; hypoglycemic drug use

## Table S6. Liner regression analysis between follow up PWV and the number of childbirths in hypertension history subgroups in Model II

| Exposure | Hypertension Yes | Hypertension No | Total |
|---|---|---|---|
| | B (95%CI) *P*-value | B (95%CI) *P*-value | B (95%CI) *P*-value |
| Age | 5.5 (-0.8, 11.9) 0.088 | 6.3 (-1.6, 14.2) 0.120 | 5.8 (0.9, 10.6) 0.020 |
| BMI | -7.9 (-14.8, -1.0) 0.026 | -6.3 (-14.5, 1.9) 0.133 | -5.9 (-11.1, -0.8) 0.025 |
| SBP | 0.4 (-1.2, 1.9) 0.643 | 0.4 (-2.4, 3.2) 0.767 | 0.3 (-1.0, 1.6) 0.610 |
| DBP | 0.0 (-1.4, 1.4) 0.994 | 1.4 (-2.1, 4.9) 0.445 | 0.0 (-1.2, 1.2) 0.977 |
| TG | -0.8 (-46.1, 44.4) 0.972 | 25.4 (-31.3, 82.0) 0.381 | 4.6 (-30.0, 39.2) 0.796 |
| GGT | -1.0 (-2.2, 0.2) 0.103 | 0.3 (-1.1, 1.8) 0.652 | -0.6 (-1.5, 0.3) 0.206 |
| LDL_C | -104.1 (-243.0, 34.8) 0.142 | 80.1 (-99.2, 259.5) 0.382 | -42.9 (-150.5, 64.6) 0.434 |
| HDL_C | -85.6 (-224.3, 53.0) 0.227 | 82.7 (-91.1, 256.5) 0.352 | -38.5 (-144.2, 67.2) 0.475 |
| FBG | 1.8 (-21.8, 25.3) 0.884 | -8.8 (-32.9, 15.3) 0.474 | -7.2 (-23.6, 9.1) 0.385 |
| PBG | 3.3 (-4.6, 11.2) 0.410 | 8.2 (-2.0, 18.5) 0.115 | 5.8 (-0.3, 12.0) 0.063 |
| HbA1c | -8.2 (-49.0, 32.5) 0.692 | 14.2 (-28.5, 56.9) 0.515 | -1.1 (-29.5, 27.3) 0.940 |
| Baseline PWV | 0.4 (0.3, 0.4) <0.001 | 0.5 (0.4, 0.6) <0.001 | 0.4 (0.4, 0.5) <0.001 |
| The number of childbirths | | | |
| 1 | 0 | 0 | 0 |
| 2 | 54.1 (-14.4, 122.6) 0.122 | 35.2 (-32.2, 102.5) 0.307 | 45.4 (-2.5, 93.4) 0.064 |
| 3 | 66.0 (-37.0, 169.0) 0.210 | 27.6 (-82.6, 137.9) 0.623 | 56.3 (-17.6, 130.2) 0.136 |
| CHD history | | | |
| Yes | 0 | 0 | 0 |
| No | -38.6 (-96.3, 19.0) 0.190 | -110.0 (-201.7, -18.4) 0.019 | -48.5 (-95.0, -1.9) 0.042 |
| hyperlipidemia history | | | |
| Yes | 0 | 0 | 0 |
| No | 38.4 (-10.9, 87.6) 0.127 | -22.4 (-76.7, 32.0) 0.420 | 4.3 (-31.6, 40.1) 0.815 |
| age at first gestation | -0.6 (-6.6, 5.5) 0.853 | 1.4 (-5.9, 8.8) 0.700 | -0.1 (-4.7, 4.5) 0.972 |
| age at menopause | 0.5 (-2.0, 2.9) 0.702 | -0.5 (-2.9, 1.8) 0.649 | -0.0 (-1.7, 1.6) 0.955 |
| number of pregnancies | | | |
| 0 | 0 | 0 | 0 |
| 1 | 0 | 0 | 0 |
| 2 | 19.5 (-60.9, 100.0) 0.634 | -22.1 (-93.4, 49.1) 0.543 | -0.2 (-53.9, 53.6) 0.995 |
| 3 | 1.2 (-82.6, 85.0) 0.977 | -38.4 (-115.4, 38.7) 0.330 | -18.9 (-75.3, 37.5) 0.511 |
| 4 | 11.7 (-84.9, 108.3) 0.813 | -90.9 (-183.0, 1.1) 0.054 | -32.3 (-98.6, 34.0) 0.340 |
| 5 | 14.4 (-101.2, 130.0) 0.808 | -1.1 (-148.2, 146.0) 0.989 | 12.0 (-74.9, 98.9) 0.787 |
| 6 | 11.7 (-182.9, 206.2) 0.906 | 89.4 (-169.6, 348.4) 0.499 | 19.6 (-130.1, 169.3) 0.798 |
| 8 | -311.3 (-628.0, 5.4) 0.054 | 0 | -329.0 (-624.4, -33.6) 0.029 |
| smoking status | | | |
| No | 0 | 0 | 0 |
| Occasional smokers | -107.1 (-332.3, 118.1) 0.352 | 86.0 (-130.0, 302.0) 0.436 | 8.7 (-144.8, 162.3) 0.911 |

| | | | |
|---|---|---|---|
| Regular smokers | -90.9 (-290.3, 108.4) 0.372 | 9.5 (-124.9, 143.9) 0.890 | -17.2 (-128.8, 94.4) 0.763 |
| drinking status | | | |
| No | 0 | 0 | 0 |
| Occasional drinkers | 5.1 (-90.1, 100.4) 0.916 | -72.2 (-149.6, 5.1) 0.068 | -31.5 (-92.0, 29.0) 0.307 |
| Regular drinkers | -11.4 (-176.2, 153.4) 0.892 | 125.2 (-43.5, 293.9) 0.146 | 20.6 (-93.5, 134.7) 0.724 |
| antihypertensive drug use | | | |
| Yes | 0 | 0 | 0 |
| No | -8.7 (-75.8, 58.3) 0.799 | 91.3 (-16.5, 199.2) 0.098 | 2.2 (-52.7, 57.2) 0.937 |
| TC | 83.5 (-44.1, 211.1) 0.200 | -83.3 (-249.2, 82.6) 0.326 | 31.9 (-67.3, 131.1) 0.529 |
| HDCP | | | |
| Yes | 0 | 0 | 0 |
| No | -19.4 (-93.9, 55.1) 0.610 | -65.9 (-187.4, 55.5) 0.288 | -31.7 (-93.1, 29.6) 0.311 |
| GDM | | | |
| Yes | 0 | 0 | 0 |
| No | -45.5 (-578.2, 487.1) 0.867 | 248.1 (-110.5, 606.7) 0.176 | 82.0 (-215.6, 379.6) 0.589 |
| occupation | | | |
| Worker | 0 | 0 | 0 |
| Famer | 129.5 (-404.0, 662.9) 0.635 | 0 | 120.2 (-342.8, 583.2) 0.611 |
| Cadre | 0 | 8.3 (-696.0, 712.7) 0.981 | -0.6 (-619.9, 618.8) 0.999 |
| Office and technical, personnel, doctor, teacher | 239.0 (-227.9, 706.0) 0.316 | -86.4 (-669.3, 496.6) 0.772 | 122.5 (-285.4, 530.5) 0.556 |
| Service worker | 0 | 0 | 0 |
| Housewife | -52.2 (-458.1, 353.7) 0.801 | 22.5 (-546.4, 591.5) 0.938 | -34.3 (-412.3, 343.6) 0.859 |
| Unemployed | -33.5 (-413.5, 346.5) 0.863 | -123.2 (-632.9, 386.4) 0.636 | -36.5 (-395.2, 322.2) 0.842 |
| Other | 68.8 (-381.6, 519.2) 0.765 | -93.1 (-673.2, 486.9) 0.753 | 22.1 (-379.7, 423.8) 0.914 |
| Education | | | |
| Illiterate | 0 | 0 | 0 |
| Primary school | 64.8 (-58.8, 188.4) 0.304 | 127.6 (-36.5, 291.7) 0.128 | 75.1 (-20.2, 170.4) 0.123 |
| Junior high school | 73.9 (-48.0, 195.8) 0.235 | 64.2 (-93.1, 221.6) 0.424 | 60.8 (-32.5, 154.1) 0.202 |
| Senior high school | 53.1 (-74.4, 180.5) 0.415 | 90.3 (-69.2, 249.9) 0.268 | 55.4 (-40.8, 151.6) 0.259 |
| College | 27.6 (-102.4, 157.6) 0.678 | 80.9 (-82.9, 244.7) 0.334 | 44.2 (-54.7, 143.2) 0.381 |
| Marriage status | | | |
| Married | 0 | 0 | 0 |
| Widowed | 0 | 0 | 0 |
| Separated | -35.0 (-104.9, 34.9) 0.327 | 105.3 (14.9, 195.7) 0.023 | 10.6 (-43.3, 64.5) 0.700 |
| Divorced | -327.2 (-871.4, 217.0) 0.239 | 0 | -290.2 (-800.3, 219.9) 0.265 |

Adjusted model II adjusted for adjusted model I + TC; hypertensive disorder complicating pregnancy (HDCP); gestational diabetes mellitus (GDM)

**Table S7. Logistical regression analysis between change in PWV as a category variable in hypertension subgroups in Model II**

| Exposure | Hypertension yes | Hypertension No | Total |
|---|---|---|---|
| | OR (95%CI) *P*-value | OR (95%CI) *P*-value | OR (95%CI) *P*-value |
| Age | 1.0 (0.9, 1.1) 0.996 | 1.0 (0.9, 1.1) 0.749 | 1.0 (1.0, 1.0) 0.799 |
| BMI | 1.0 (0.9, 1.0) 0.280 | 1.0 (0.9, 1.1) 0.814 | 1.0 (0.9, 1.0) 0.637 |
| SBP | 1.0 (1.0, 1.0) 0.042 | 1.0 (0.9, 1.0) 0.002 | 1.0 (1.0, 1.0) 0.003 |
| DBP | 1.0 (1.0, 1.0) 0.319 | 1.0 (1.0, 1.1) 0.115 | 1.0 (1.0, 1.0) 0.235 |
| TG | 0.9 (0.6, 1.4) 0.670 | 1.3 (0.7, 2.3) 0.371 | 1.1 (0.8, 1.5) 0.692 |
| GGT | 1.0 (1.0, 1.0) 0.154 | 1.0 (1.0, 1.0) 0.656 | 1.0 (1.0, 1.0) 0.258 |
| LDL_C | 0.3 (0.1, 1.1) 0.076 | 2.9 (0.5, 17.1) 0.248 | 0.8 (0.3, 2.1) 0.632 |
| HDL_C | 0.4 (0.1, 1.3) 0.110 | 3.0 (0.5, 17.0) 0.206 | 0.9 (0.3, 2.2) 0.747 |
| FBG | 1.0 (0.8, 1.3) 0.915 | 0.8 (0.6, 1.1) 0.221 | 0.9 (0.8, 1.1) 0.231 |
| PBG | 1.0 (0.9, 1.1) 0.514 | 1.1 (1.0, 1.2) 0.153 | 1.0 (0.9, 1.1) 0.824 |
| HbA1c | 0.9 (0.6, 1.3) 0.500 | 1.1 (0.7, 1.7) 0.570 | 1.0 (0.8, 1.3) 0.966 |
| The number of childbirths | | | |
| 1 | 1.0 | 1.0 | 1.0 |
| 2 | 1.9 (1.0, 3.7) 0.052 | 1.1 (0.6, 2.1) 0.800 | 1.4 (0.9, 2.2) 0.124 |
| 3 | 1.7 (0.6, 4.4) 0.305 | 1.3 (0.4, 3.8) 0.690 | 1.6 (0.8, 3.1) 0.195 |
| CHD history | | | |
| Yes | 1.0 | 1.0 | 1.0 |
| No | 0.8 (0.5, 1.4) 0.507 | 0.6 (0.2, 1.4) 0.246 | 0.9 (0.6, 1.3) 0.504 |
| hyperlipidemia history | | | |
| Yes | 1.0 | 1.0 | 1.0 |
| No | 1.0 (0.6, 1.6) 0.966 | 0.6 (0.4, 1.1) 0.079 | 0.8 (0.6, 1.2) 0.275 |
| age at first gestation | 1.0 (1.0, 1.1) 0.654 | 1.0 (0.9, 1.1) 0.614 | 1.0 (1.0, 1.1) 0.430 |
| age at menopause | 1.0 (1.0, 1.0) 0.762 | 1.0 (1.0, 1.0) 0.560 | 1.0 (1.0, 1.0) 0.952 |
| number of pregnancies | | | |
| 0 | 1.0 | 1.0 | 1.0 |
| 1 | 1.0 | 1.0 | 1.0 |
| 2 | 0.7 (0.3, 1.6) 0.405 | 0.9 (0.5, 1.9) 0.839 | 0.8 (0.5, 1.4) 0.503 |
| 3 | 0.6 (0.3, 1.4) 0.257 | 0.9 (0.4, 1.9) 0.786 | 0.7 (0.4, 1.3) 0.277 |
| 4 | 0.7 (0.3, 1.9) 0.521 | 0.5 (0.2, 1.3) 0.152 | 0.6 (0.3, 1.2) 0.173 |
| 5 | 1.1 (0.4, 3.2) 0.834 | 1.3 (0.3, 5.5) 0.727 | 1.1 (0.5, 2.5) 0.735 |
| 6 | 1.9 (0.4, 10.0) 0.433 | 2.8 (0.2, 33.8) 0.421 | 2.0 (0.6, 7.2) 0.269 |
| 8 | 0.0 (0.0, Inf) 0.985 | 1.0 | 0.0 (0.0, Inf) 0.978 |
| smoking status | | | |
| No | 1.0 | 1.0 | 1.0 |
| Occasional smokers | 1.1 (0.2, 7.1) 0.927 | 0.8 (0.1, 6.8) 0.838 | 1.5 (0.4, 5.4) 0.552 |
| Regular smokers | 0.6 (0.1, 5.5) 0.650 | 1.4 (0.4, 5.2) 0.641 | 1.0 (0.4, 2.8) 0.994 |
| drinking status | | | |
| No | 1.0 | 1.0 | 1.0 |
| Occasional drinkers | 1.6 (0.7, 3.7) 0.258 | 0.7 (0.3, 1.7) 0.442 | 1.0 (0.6, 1.8) 0.882 |
| Regular drinkers | 0.5 (0.1, 2.9) 0.447 | 1.0 (0.2, 6.0) 0.966 | 0.9 (0.3, 2.6) 0.788 |

| | | | |
|---|---|---|---|
| **hypoglycemic drug use** | | | |
| **Yes** | 1.0 | 1.0 | 1.0 |
| **No** | 0.6 (0.3, 1.2) 0.167 | 0.9 (0.3, 2.7) 0.913 | 0.7 (0.4, 1.2) 0.261 |
| **TC** | 2.4 (0.8, 7.2) 0.105 | 0.3 (0.1, 1.8) 0.211 | 1.2 (0.5, 2.8) 0.758 |
| **HDCP** | | | |
| **Yes** | 1.0 | 1.0 | 1.0 |
| **No** | 0.8 (0.4, 1.6) 0.601 | 0.6 (0.2, 1.9) 0.387 | 0.8 (0.5, 1.4) 0.476 |
| **GDM** | | | |
| **Yes** | 1.0 | 1.0 | 1.0 |
| **No** | 1953436.6 (0.0, Inf) 0.992 | 0.6 (0.0, 15.4) 0.758 | 0.8 (0.1, 10.7) 0.893 |
| **occupation** | | | |
| **Worker** | 1.0 | 1.0 | 1.0 |
| **Famer** | 0.3 (0.0, 21.8) 0.608 | 1.0 | 0.7 (0.0, 36.5) 0.880 |
| **Cadre** | 1.0 | 0.2 (0.0, Inf) 0.999 | 536376.7 (0.0, Inf) 0.988 |
| **Office and technical, personnel, doctor, teacher** | 0.6 (0.0, 24.1) 0.790 | 0.0 (0.0, Inf) 0.985 | 0.3 (0.0, 8.1) 0.448 |
| **Service worker** | 1.0 | 1.0 | 1.0 |
| **Housewife** | 0.1 (0.0, 3.6) 0.231 | 0.0 (0.0, Inf) 0.986 | 0.1 (0.0, 3.2) 0.209 |
| **Unemployed** | 0.1 (0.0, 2.5) 0.175 | 0.0 (0.0, Inf) 0.985 | 0.1 (0.0, 2.4) 0.168 |
| **Other** | 0.1 (0.0, 3.2) 0.174 | 0.0 (0.0, Inf) 0.986 | 0.1 (0.0, 3.0) 0.182 |
| **Education** | | | |
| **Illiterate** | 1.0 | 1.0 | 1.0 |
| **Primary school** | 1.2 (0.4, 3.7) 0.733 | 2.8 (0.3, 23.6) 0.339 | 1.6 (0.6, 4.0) 0.348 |
| **Junior high school** | 0.7 (0.2, 2.3) 0.593 | 2.9 (0.4, 22.9) 0.310 | 1.1 (0.4, 2.8) 0.821 |
| **Senior high school** | 0.7 (0.2, 2.2) 0.508 | 5.0 (0.6, 40.4) 0.129 | 1.4 (0.5, 3.6) 0.485 |
| **College** | 0.6 (0.2, 1.9) 0.350 | 4.1 (0.5, 34.3) 0.191 | 1.2 (0.4, 3.1) 0.754 |
| **Marriage status** | | | |
| **Married** | 1.0 | 1.0 | 1.0 |
| **Widowed** | 1.0 | 1.0 | 1.0 |
| **Separated** | 0.6 (0.3, 1.2) 0.121 | 3.0 (1.2, 7.1) 0.014 | 1.1 (0.6, 1.8) 0.779 |
| **Divorced** | 0.0 (0.0, Inf) 0.991 | 1.0 | 0.0 (0.0, Inf) 0.987 |

Adjusted model II adjusted for adjusted model I + TC; hypertensive disorder complicating pregnancy (HDCP); gestational diabetes mellitus (GDM)

## Table S8. Logistical regression analysis between change in PWV as a category variable in hypertension subgroups in Model I

| Exposure | Hypertension Yes | Hypertension No | Total |
|---|---|---|---|
| | OR (95%CI) *P*-value | OR (95%CI) *P*-value | OR (95%CI) *P*-value |
| Age | 1.0 (1.0, 1.1) 0.889 | 1.0 (0.9, 1.1) 0.791 | 1.0 (1.0, 1.0) 0.859 |
| BMI | 1.0 (0.9, 1.0) 0.489 | 1.0 (0.9, 1.1) 0.694 | 1.0 (0.9, 1.0) 0.707 |
| SBP | 1.0 (1.0, 1.0) 0.050 | 1.0 (0.9, 1.0) 0.002 | 1.0 (1.0, 1.0) 0.003 |
| DBP | 1.0 (1.0, 1.0) 0.327 | 1.0 (1.0, 1.1) 0.161 | 1.0 (1.0, 1.0) 0.260 |
| TG | 1.1 (0.9, 1.4) 0.444 | 1.0 (0.7, 1.3) 0.986 | 1.1 (0.9, 1.3) 0.379 |
| GGT | 1.0 (1.0, 1.0) 0.191 | 1.0 (1.0, 1.0) 0.814 | 1.0 (1.0, 1.0) 0.298 |
| LDL_C | 0.9 (0.7, 1.1) 0.308 | 0.9 (0.7, 1.2) 0.565 | 0.9 (0.8, 1.1) 0.300 |
| HDL_C | 0.8 (0.4, 1.5) 0.420 | 1.2 (0.6, 2.5) 0.588 | 1.0 (0.6, 1.6) 0.883 |
| FBG | 1.0 (0.8, 1.2) 0.761 | 0.9 (0.7, 1.1) 0.299 | 0.9 (0.8, 1.1) 0.237 |
| PBG | 1.0 (0.9, 1.1) 0.666 | 1.1 (1.0, 1.2) 0.251 | 1.0 (1.0, 1.1) 0.791 |
| HbA1c | 0.9 (0.6, 1.3) 0.624 | 1.1 (0.7, 1.6) 0.599 | 1.0 (0.8, 1.3) 0.933 |
| The number of childbirths | | | |
| 1 | 1.0 | 1.0 | 1.0 |
| 2 | 1.8 (1.0, 3.5) 0.060 | 1.1 (0.6, 2.2) 0.698 | 1.4 (0.9, 2.2) 0.107 |
| 3 | 1.9 (0.7, 4.8) 0.197 | 1.3 (0.4, 3.9) 0.613 | 1.6 (0.8, 3.1) 0.189 |
| CHD history | | | |
| Yes | 1.0 | 1.0 | 1.0 |
| No | 0.9 (0.5, 1.5) 0.661 | 0.6 (0.3, 1.4) 0.256 | 0.9 (0.6, 1.3) 0.522 |
| hyperlipidemia history | | | |
| Yes | 1.0 | 1.0 | 1.0 |
| No | 1.0 (0.6, 1.6) 0.990 | 0.6 (0.4, 1.0) 0.070 | 0.8 (0.6, 1.1) 0.260 |
| age at first gestation | 1.0 (1.0, 1.1) 0.713 | 1.0 (1.0, 1.1) 0.411 | 1.0 (1.0, 1.1) 0.437 |
| age at menopause | 1.0 (1.0, 1.0) 0.889 | 1.0 (1.0, 1.0) 0.724 | 1.0 (1.0, 1.0) 0.958 |
| number of pregnancies | | | |
| 0 | 1.0 | 1.0 | 1.0 |
| 1 | 1.0 | 1.0 | 1.0 |
| 2 | 0.8 (0.4, 1.7) 0.563 | 0.9 (0.5, 1.7) 0.737 | 0.9 (0.5, 1.4) 0.515 |
| 3 | 0.7 (0.3, 1.5) 0.348 | 0.9 (0.5, 1.9) 0.851 | 0.8 (0.5, 1.3) 0.343 |
| 4 | 0.8 (0.3, 1.9) 0.579 | 0.5 (0.2, 1.3) 0.141 | 0.7 (0.4, 1.2) 0.200 |
| 5 | 1.2 (0.4, 3.2) 0.785 | 1.1 (0.3, 4.4) 0.911 | 1.1 (0.5, 2.4) 0.771 |
| 6 | 1.7 (0.3, 8.4) 0.508 | 2.6 (0.3, 24.5) 0.404 | 1.9 (0.6, 6.6) 0.305 |
| 8 | 0.0 (0.0, Inf) 0.978 | 1.0 | 0.0 (0.0, Inf) 0.978 |
| smoking status | | | |
| No | 1.0 | 1.0 | 1.0 |
| Occasional smokers | 1.3 (0.2, 7.7) 0.805 | 1.0 (0.1, 7.2) 0.969 | 1.4 (0.4, 5.0) 0.611 |
| Regular smokers | 0.6 (0.1, 5.0) 0.605 | 1.4 (0.4, 4.8) 0.600 | 1.0 (0.4, 2.7) 0.952 |
| drinking status | | | |
| No | 1.0 | 1.0 | 1.0 |
| Occasional drinkers | 1.6 (0.7, 3.7) 0.229 | 0.8 (0.3, 1.6) 0.476 | 1.0 (0.6, 1.8) 0.889 |

| | | | |
|---|---|---|---|
| **Regular drinkers** | 0.6 (0.1, 3.4) 0.603 | 1.2 (0.3, 5.0) 0.847 | 1.0 (0.3, 2.8) 0.930 |
| **hypoglycemic drug use** | | | |
| **Yes** | 1.0 | 1.0 | 1.0 |
| **No** | 0.6 (0.3, 1.2) 0.144 | 1.0 (0.4, 2.6) 0.929 | 0.7 (0.4, 1.2) 0.179 |

Adjusted model I adjusted for age; BMI; SBP; DBP; TG; GGT; LDL-C; HDL-C; FBG; PBG; HbA1c ; CHD history; hyperlipidemia history; age at first gestation; age at menopause; number of pregnancies; smoking status; drinking status; antihypertensive drug use; hypoglycemic drug use

## Table S9. Liner regression analysis between change in PWV as a continuous variable in hypertension subgroups in Model I

| Exposure | Hypertension Yes | Hypertension No | Total |
|---|---|---|---|
| | B (95%CI) *P*-value | B (95%CI) *P*-value | B (95%CI) *P*-value |
| Age | -9.2 (-16.7, -1.8) 0.015 | -2.6 (-10.5, 5.2) 0.512 | -7.4 (-12.7, -2.0) 0.007 |
| BMI | -0.1 (-8.5, 8.4) 0.987 | -1.3 (-10.2, 7.6) 0.771 | 0.2 (-5.9, 6.3) 0.953 |
| SBP | -4.1 (-5.9, -2.2) <0.001 | -4.3 (-7.2, -1.4) 0.004 | -4.1 (-5.6, -2.6) <0.001 |
| DBP | 1.3 (-0.5, 3.0) 0.149 | 1.7 (-2.1, 5.5) 0.389 | 1.3 (-0.2, 2.7) 0.086 |
| TG | 3.8 (-32.9, 40.4) 0.841 | -6.0 (-40.9, 29.0) 0.739 | 3.8 (-21.8, 29.4) 0.771 |
| GGT | -0.8 (-2.4, 0.7) 0.291 | 0.6 (-1.0, 2.2) 0.455 | -0.4 (-1.5, 0.7) 0.467 |
| LDL_C | -10.3 (-42.7, 22.1) 0.535 | -10.8 (-42.0, 20.4) 0.499 | -8.3 (-31.1, 14.6) 0.480 |
| HDL_C | -17.2 (-107.4, 72.9) 0.708 | 11.1 (-71.5, 93.8) 0.792 | -4.3 (-66.0, 57.5) 0.893 |
| FBG | 1.3 (-28.5, 31.0) 0.934 | -13.3 (-39.7, 13.1) 0.325 | -8.9 (-28.7, 10.9) 0.379 |
| PBG | 1.5 (-8.5, 11.4) 0.772 | 9.0 (-2.3, 20.2) 0.119 | 4.1 (-3.3, 11.5) 0.278 |
| HbA1c | -36.7 (-87.9, 14.6) 0.161 | 12.9 (-33.1, 58.9) 0.582 | -17.5 (-51.7, 16.7) 0.316 |
| The number of childbirths | | | |
| 1 | 0 | 0 | 0 |
| 2 | 71.2 (-15.2, 157.6) 0.107 | 36.1 (-37.6, 109.7) 0.337 | 52.5 (-5.3, 110.3) 0.076 |
| 3 | 102.8 (-26.0, 231.5) 0.118 | 47.5 (-73.4, 168.4) 0.441 | 81.7 (-7.0, 170.5) 0.071 |
| CHD history | | | |
| Yes | 0 | 0 | 0 |
| No | -60.0 (-132.5, 12.5) 0.105 | -94.6 (-193.2, 4.0) 0.061 | -60.3 (-116.7, -3.9) 0.036 |
| hyperlipidemia history | | | |
| Yes | 0 | 0 | 0 |
| No | 3.3 (-57.8, 64.3) 0.917 | -23.8 (-83.1, 35.5) 0.432 | -9.3 (-52.1, 33.5) 0.669 |
| age at first gestation | -0.4 (-8.0, 7.2) 0.921 | 2.2 (-5.7, 10.1) 0.586 | -0.1 (-5.6, 5.4) 0.966 |
| age at menopause | -0.5 (-3.6, 2.6) 0.737 | -0.4 (-3.1, 2.2) 0.737 | -0.4 (-2.5, 1.6) 0.695 |
| number of pregnancies | | | |
| 0 | 0 | 0 | 0 |
| 1 | 0 | 0 | 0 |
| 2 | 11.0 (-90.1, 112.0) 0.832 | -23.0 (-101.3, 55.3) 0.565 | -0.7 (-65.5, 64.1) 0.984 |
| 3 | -35.2 (-140.7, 70.3) 0.513 | -29.7 (-113.7, 54.3) 0.489 | -33.0 (-101.0, 35.0) 0.342 |
| 4 | 20.8 (-100.5, 142.1) 0.737 | -57.6 (-158.9, 43.6) 0.265 | -5.1 (-85.2, 74.9) 0.900 |
| 5 | 49.8 (-94.7, 194.3) 0.500 | -50.3 (-212.5, 111.9) 0.544 | 16.4 (-88.2, 121.0) 0.758 |
| 6 | -59.1 (-304.6, 186.4) 0.637 | 167.8 (-112.5, 448.0) 0.241 | -8.5 (-189.3, 172.3) 0.927 |
| 8 | -263.1 (-666.0, 139.7) 0.201 | 0 | -252.4 (-611.5, 106.8) 0.169 |
| smoking status | | | |
| No | 0 | 0 | 0 |
| Occasional smokers | 40.8 (-242.6, 324.2) 0.778 | 126.8 (-110.9, 364.5) 0.296 | 90.6 (-95.6, 276.8) 0.341 |
| Regular smokers | 37.8 (-215.4, 291.0) 0.770 | -0.5 (-143.2, 142.3) 0.995 | 17.5 (-116.0, 150.9) 0.797 |
| drinking status | | | |
| No | 0 | 0 | 0 |
| Occasional drinkers | 61.7 (-57.9, 181.2) 0.312 | -58.2 (-142.8, 26.5) 0.179 | -6.8 (-79.5, 66.0) 0.855 |

| | | | |
|---|---|---|---|
| **Regular drinkers** | -65.2 (-274.4, 144.1) 0.542 | 116.2 (-58.7, 291.2) 0.194 | 14.9 (-122.2, 152.1) 0.831 |
| **hypoglycemic drug use** | | | |
| **Yes** | 0 | 0 | 0 |
| **No** | -74.8 (-159.1, 9.5) 0.083 | 146.1 (31.5, 260.7) 0.013 | -22.6 (-88.7, 43.6) 0.503 |

Adjusted model I adjusted for age; BMI; SBP; DBP; TG; GGT; LDL-C; HDL-C; FBG; PBG; HbA1c; CHD history; hyperlipidemia history; age at first gestation; age at menopause; number of pregnancies; smoking status; drinking status; antihypertensive drug use; hypoglycemic drug use

## Table S10. Liner regression analysis between change in PWV as a continuous variable in hypertension subgroups in Model II

| Exposure | Hypertension Yes | Hypertension No | Total |
|---|---|---|---|
| | B (95%CI) *P*-value | B (95%CI) *P*-value | B (95%CI) *P*-value |
| Age | -9.8 (-17.7, -1.8) 0.016 | -4.5 (-13.0, 4.0) 0.301 | -8.2 (-13.9, -2.5) 0.005 |
| BMI | -1.6 (-10.5, 7.3) 0.722 | -2.3 (-11.4, 6.9) 0.628 | -0.4 (-6.7, 5.9) 0.904 |
| SBP | -4.1 (-6.0, -2.3) <0.001 | -4.0 (-7.0, -1.1) 0.008 | -4.1 (-5.6, -2.6) <0.001 |
| DBP | 1.4 (-0.4, 3.2) 0.127 | 1.9 (-2.0, 5.8) 0.338 | 1.3 (-0.2, 2.8) 0.080 |
| TG | -6.5 (-64.9, 51.8) 0.826 | 23.2 (-40.0, 86.5) 0.472 | 4.5 (-38.0, 46.9) 0.837 |
| GGT | -0.8 (-2.4, 0.7) 0.289 | 0.7 (-1.0, 2.3) 0.423 | -0.4 (-1.6, 0.7) 0.445 |
| LDL_C | -82.8 (-261.9, 96.4) 0.366 | 120.4 (-79.6, 320.4) 0.239 | -15.8 (-147.8, 116.1) 0.814 |
| HDL_C | -75.8 (-254.7, 103.1) 0.407 | 119.5 (-74.4, 313.4) 0.228 | -10.4 (-140.1, 119.2) 0.875 |
| FBG | 2.5 (-27.8, 32.8) 0.872 | -11.1 (-38.0, 15.8) 0.419 | -9.0 (-29.0, 11.0) 0.380 |
| PBG | 2.0 (-8.2, 12.2) 0.699 | 8.3 (-3.2, 19.7) 0.157 | 4.3 (-3.2, 11.9) 0.259 |
| HbA1c | -43.6 (-96.0, 8.8) 0.103 | 17.0 (-30.6, 64.7) 0.484 | -19.4 (-54.2, 15.3) 0.273 |
| The number of childbirths | | | |
| 1 | 0 | 0 | 0 |
| 2 | 77.0 (-11.3, 165.4) 0.088 | 35.8 (-39.4, 110.9) 0.352 | 55.0 (-3.9, 113.8) 0.067 |
| 3 | 96.2 (-36.6, 229.1) 0.156 | 46.8 (-76.3, 169.8) 0.457 | 83.5 (-7.1, 174.1) 0.071 |
| CHD history | | | |
| Yes | 0 | 0 | 0 |
| No | -70.7 (-144.9, 3.6) 0.063 | -98.2 (-200.5, 4.1) 0.061 | -64.3 (-121.5, -7.2) 0.028 |
| hyperlipidemia history | | | |
| Yes | 0 | 0 | 0 |
| No | 9.7 (-53.7, 73.2) 0.764 | -24.6 (-85.2, 36.1) 0.427 | -10.6 (-54.5, 33.3) 0.636 |
| age at first gestation | -0.0 (-7.8, 7.8) 1.000 | 0.9 (-7.3, 9.1) 0.826 | 0.4 (-5.3, 6.0) 0.895 |
| age at menopause | -0.4 (-3.5, 2.8) 0.808 | -0.4 (-3.1, 2.2) 0.756 | -0.4 (-2.5, 1.6) 0.678 |
| number of pregnancies | | | |
| 0 | 0 | 0 | 0 |
| 1 | 0 | 0 | 0 |
| 2 | 17.0 (-86.8, 120.7) 0.749 | -19.2 (-98.7, 60.4) 0.637 | 2.2 (-63.7, 68.2) 0.947 |
| 3 | -33.8 (-141.9, 74.3) 0.540 | -31.4 (-117.4, 54.7) 0.475 | -32.1 (-101.3, 37.1) 0.363 |
| 4 | 23.7 (-100.9, 148.3) 0.709 | -60.9 (-163.4, 41.7) 0.246 | -6.2 (-87.5, 75.1) 0.882 |
| 5 | 52.0 (-97.0, 201.1) 0.494 | -36.8 (-200.9, 127.2) 0.660 | 21.2 (-85.3, 127.8) 0.696 |
| 6 | -57.6 (-308.4, 193.3) 0.653 | 171.1 (-117.5, 459.6) 0.246 | -7.1 (-190.8, 176.5) 0.939 |
| 8 | -277.4 (-685.9, 131.1) 0.184 | 0 | -261.0 (-623.3, 101.4) 0.158 |
| smoking status | | | |
| No | 0 | 0 | 0 |
| Occasional smokers | 31.6 (-258.4, 321.6) 0.831 | 99.3 (-141.9, 340.4) 0.420 | 91.0 (-97.2, 279.2) 0.344 |
| Regular smokers | 16.3 (-240.5, 273.2) 0.901 | 22.7 (-127.3, 172.7) 0.767 | 24.3 (-112.6, 161.1) 0.728 |
| drinking status | | | |
| No | 0 | 0 | 0 |
| Occasional drinkers | 49.6 (-73.2, 172.3) 0.429 | -57.6 (-143.9, 28.7) 0.192 | -5.7 (-79.9, 68.4) 0.879 |
| Regular drinkers | -67.1 (-279.6, 145.4) 0.536 | 108.6 (-79.8, 296.9) 0.259 | 1.6 (-138.4, 141.6) 0.982 |

| | | | |
|---|---|---|---|
| hypoglycemic drug use | | | |
| Yes | 0 | 0 | 0 |
| No | -79.9 (-165.9, 6.1) 0.069 | 165.7 (46.5, 285.0) 0.007 | -19.6 (-87.0, 47.8) 0.569 |
| TC | 64.1 (-100.4, 228.7) 0.445 | -122.9 (-307.9, 62.1) 0.194 | 6.4 (-115.2, 128.0) 0.918 |
| HDCP | | | |
| Yes | 0 | 0 | 0 |
| No | 70.6 (-24.8, 166.1) 0.147 | -33.4 (-168.8, 102.0) 0.629 | 37.0 (-37.9, 112.0) 0.333 |
| GDM | | | |
| Yes | 0 | 0 | 0 |
| No | -213.8 (-900.6, 473.1) 0.542 | 327.8 (-72.2, 727.8) 0.109 | 57.4 (-307.7, 422.5) 0.758 |
| occupation | | | |
| Worker | 0 | 0 | 0 |
| Famer | -1.3 (-689.3, 686.7) 0.997 | 0 | 111.6 (-456.4, 679.6) 0.700 |
| Cadre | 0 | -47.2 (-833.4, 739.0) 0.906 | 78.2 (-681.6, 838.1) 0.840 |
| Office and technical, personnel, doctor, teacher | 148.7 (-453.7, 751.0) 0.629 | -321.6 (-970.3, 327.1) 0.332 | 34.1 (-466.3, 534.5) 0.894 |
| Service worker | 0 | 0 | 0 |
| Housewife | -179.7 (-703.1, 343.7) 0.501 | -244.7 (-877.1, 387.7) 0.449 | -147.3 (-610.9, 316.3) 0.534 |
| Unemployed | -106.2 (-596.4, 384.0) 0.671 | -322.5 (-889.8, 244.8) 0.266 | -86.1 (-526.2, 354.0) 0.701 |
| Other | -227.8 (-807.7, 352.0) 0.442 | -214.0 (-861.0, 433.0) 0.517 | -118.4 (-611.1, 374.3) 0.638 |
| Education | | | |
| Illiterate | 0 | 0 | 0 |
| Primary school | -0.1 (-159.3, 159.1) 0.999 | 165.9 (-17.1, 349.0) 0.076 | 39.8 (-77.1, 156.6) 0.505 |
| Junior high school | -19.6 (-176.4, 137.3) 0.807 | 102.9 (-72.5, 278.4) 0.251 | 10.7 (-103.6, 125.0) 0.855 |
| Senior high school | -58.3 (-222.0, 105.5) 0.486 | 134.0 (-43.9, 311.8) 0.141 | 0.8 (-117.1, 118.8) 0.989 |
| College | -58.8 (-226.2, 108.6) 0.491 | 139.2 (-43.2, 321.7) 0.136 | 1.6 (-119.8, 122.9) 0.980 |
| Marriage status | | | |
| Married | 0 | 0 | 0 |
| Widowed | 0 | 0 | 0 |
| Separated | -20.0 (-110.1, 70.2) 0.665 | 113.7 (12.9, 214.6) 0.028 | 23.7 (-42.4, 89.9) 0.482 |
| Divorced | -294.6 (-996.7, 407.4) 0.411 | 0 | -253.7 (-879.5, 372.1) 0.427 |

Adjusted model II adjusted for adjusted model I + TC; hypertensive disorder complicating pregnancy (HDCP); gestational diabetes mellitus (GDM)

## Table S11. Liner regression analysis between follow up PWV and the number of childbirths in diabetes history subgroups in Model I

| Exposure | Diabetes Yes<br>B (95%CI) *P*-value | Diabetes No<br>B (95%CI) *P*-value | Total<br>B (95%CI) *P*-value |
|---|---|---|---|
| Age | 9.0 (2.0, 16.1) 0.013 | 2.8 (-3.3, 8.9) 0.365 | 5.7 (1.2, 10.2) 0.013 |
| BMI | -7.1 (-15.6, 1.4) 0.102 | -4.6 (-10.9, 1.7) 0.150 | -5.2 (-10.2, -0.3) 0.038 |
| SBP | -0.5 (-2.3, 1.2) 0.540 | 1.4 (-0.3, 3.0) 0.099 | 0.3 (-0.8, 1.5) 0.560 |
| DBP | 0.4 (-1.1, 1.8) 0.611 | -0.8 (-3.5, 2.0) 0.593 | 0.1 (-1.1, 1.3) 0.887 |
| TG | -8.4 (-42.6, 25.7) 0.629 | 21.0 (-6.2, 48.1) 0.130 | 10.3 (-10.5, 31.2) 0.331 |
| GGT | -1.1 (-2.7, 0.5) 0.168 | 0.1 (-1.1, 1.2) 0.907 | -0.5 (-1.4, 0.4) 0.273 |

| | | | |
|---|---|---|---|
| **LDL_C** | -6.5 (-36.4, 23.4) 0.671 | -8.0 (-32.3, 16.2) 0.517 | -9.6 (-28.2, 9.0) 0.313 |
| **HDL_C** | -6.1 (-94.6, 82.4) 0.893 | -3.6 (-66.6, 59.5) 0.912 | -12.6 (-63.0, 37.7) 0.623 |
| **FBG** | -10.1 (-28.9, 8.7) 0.295 | -3.8 (-48.5, 40.9) 0.868 | -7.6 (-23.8, 8.6) 0.358 |
| **PBG** | 6.4 (-1.5, 14.3) 0.113 | 11.5 (-1.8, 24.9) 0.091 | 7.4 (0.9, 13.9) 0.025 |
| **HbA1c** | 17.7 (-15.4, 50.8) 0.296 | -52.1 (-118.6, 14.4) 0.125 | 3.7 (-24.6, 32.1) 0.796 |
| **Baseline PWV** | 0.3 (0.3, 0.4) <0.001 | 0.5 (0.4, 0.5) <0.001 | 0.4 (0.4, 0.5) <0.001 |
| **The number of childbirths** | | | |
| **1** | 0 | 0 | 0 |
| **2** | 50.9 (-29.5, 131.4) 0.216 | 36.7 (-22.6, 96.0) 0.225 | 46.1 (-1.1, 93.2) 0.056 |
| **3** | 40.5 (-79.6, 160.6) 0.509 | 61.9 (-31.8, 155.7) 0.196 | 60.4 (-12.2, 132.9) 0.103 |
| **CHD history** | | | |
| **Yes** | 0 | 0 | 0 |
| **No** | -78.7 (-148.6, -8.9) 0.028 | -23.0 (-84.8, 38.9) 0.467 | -49.5 (-95.3, -3.8) 0.034 |
| **hyperlipidemia history** | | | |
| **Yes** | 0 | 0 | 0 |
| **No** | -21.0 (-78.7, 36.7) 0.476 | 21.5 (-23.5, 66.6) 0.350 | 3.4 (-31.5, 38.2) 0.849 |
| **age at first gestation** | 3.5 (-3.8, 10.8) 0.350 | -2.0 (-7.9, 3.9) 0.514 | 0.2 (-4.3, 4.7) 0.930 |
| **age at menopause** | 0.4 (-2.0, 2.8) 0.751 | -0.6 (-3.1, 1.8) 0.617 | 0.1 (-1.6, 1.8) 0.922 |
| **number of pregnancies** | | | |
| **0** | 0 | 0 | 0 |
| **1** | 0 | 0 | 0 |
| **2** | -10.2 (-114.6, 94.2) 0.848 | 15.3 (-46.3, 77.0) 0.626 | 0.3 (-52.7, 53.3) 0.990 |
| **3** | -3.3 (-111.8, 105.2) 0.953 | -7.6 (-73.2, 58.0) 0.820 | -20.0 (-75.6, 35.7) 0.482 |
| **4** | -33.8 (-155.5, 87.8) 0.586 | -16.2 (-95.6, 63.2) 0.689 | -33.2 (-98.6, 32.3) 0.321 |
| **5** | 38.5 (-109.0, 186.0) 0.609 | 5.9 (-103.1, 114.9) 0.915 | 9.1 (-76.3, 94.5) 0.834 |
| **6** | 26.8 (-193.5, 247.0) 0.812 | -21.3 (-234.2, 191.6) 0.845 | 9.6 (-137.9, 157.1) 0.898 |
| **8** | -344.6 (-660.4, -28.8) 0.033 | 0 | -317.1 (-609.8, -24.4) 0.034 |
| **smoking status** | | | |
| **No** | 0 | 0 | 0 |
| **Occasional smokers** | 121.7 (-101.5, 344.8) 0.286 | -130.3 (-349.0, 88.4) 0.243 | 13.3 (-139.1, 165.6) 0.864 |
| **Regular smokers** | -51.3 (-275.2, 172.6) 0.654 | 14.6 (-113.9, 143.2) 0.823 | -22.9 (-134.2, 88.3) 0.686 |
| **drinking status** | | | |
| **No** | 0 | 0 | 0 |
| **Occasional drinkers** | -137.2 (-251.1, -23.3) 0.019 | 17.1 (-53.3, 87.4) 0.635 | -30.1 (-90.0, 29.7) 0.324 |
| **Regular drinkers** | 50.2 (-137.9, 238.4) 0.601 | 2.4 (-141.2, 146.1) 0.973 | 31.1 (-81.0, 143.1) 0.587 |
| **antihypertensive drug use** | | | |
| **Yes** | 0 | 0 | 0 |
| **No** | -55.0 (-121.7, 11.8) 0.107 | 14.2 (-44.6, 73.0) 0.636 | -17.7 (-61.1, 25.6) 0.423 |

Adjusted model I adjusted for age; BMI; SBP; DBP; TG; LDL-C; HDL-C; FBG; PBG; HbA1c; baseline PWV; CHD history; hyperlipidemia history; age at first gestation; age at menopause; number of pregnancies; smoking status; drinking status; antihypertensive drug use; hypoglycemic drug use

**Table S12. Liner regression analysis between follow up PWV and the number of childbirths in diabetes history subgroups in Model II**

| Exposure | Diabetes Yes | Diabetes No | Total |
|---|---|---|---|
| | B (95%CI) *P*-value | B (95%CI) *P*-value | B (95%CI) *P*-value |
| Age | 8.4 (0.5, 16.3) 0.038 | 4.1 (-2.3, 10.5) 0.208 | 6.0 (1.1, 10.9) 0.016 |
| BMI | -7.3 (-16.6, 1.9) 0.120 | -5.4 (-11.8, 1.0) 0.101 | -5.7 (-10.8, -0.6) 0.028 |
| SBP | -0.3 (-2.1, 1.6) 0.760 | 1.7 (-0.1, 3.4) 0.060 | 0.4 (-0.8, 1.7) 0.499 |
| DBP | 0.1 (-2.6, 2.9) 0.926 | -1.2 (-4.1, 1.7) 0.425 | 0.0 (-1.7, 1.8) 0.964 |
| TG | -23.3 (-77.2, 30.5) 0.396 | 43.2 (-5.3, 91.7) 0.081 | 4.9 (-29.6, 39.3) 0.782 |
| GGT | -1.2 (-2.8, 0.4) 0.141 | -0.0 (-1.1, 1.1) 0.987 | -0.6 (-1.5, 0.3) 0.219 |
| LDL_C | -80.1 (-238.6, 78.5) 0.323 | 54.6 (-100.0, 209.2) 0.489 | -40.0 (-147.4, 67.4) 0.466 |
| HDL_C | -63.9 (-230.8, 103.0) 0.454 | 58.9 (-87.1, 204.9) 0.429 | -37.4 (-142.9, 68.0) 0.487 |
| FBG | -10.8 (-30.2, 8.5) 0.274 | -3.0 (-48.5, 42.4) 0.896 | -7.2 (-23.6, 9.1) 0.387 |
| PBG | 5.9 (-2.3, 14.2) 0.160 | 13.8 (0.2, 27.4) 0.047 | 7.3 (0.7, 13.9) 0.030 |
| HbA1c | 18.6 (-15.9, 53.1) 0.291 | -54.4 (-121.5, 12.7) 0.113 | 2.3 (-26.5, 31.1) 0.874 |
| Baseline PWV | 0.3 (0.3, 0.4) <0.001 | 0.5 (0.4, 0.5) <0.001 | 0.4 (0.4, 0.5) <0.001 |
| The number of childbirths | | | |
| 1 | 0 | 0 | 0 |
| 2 | 46.0 (-37.3, 129.3) 0.280 | 33.0 (-27.4, 93.3) 0.285 | 45.0 (-2.9, 92.9) 0.066 |
| 3 | 36.2 (-88.5, 160.9) 0.569 | 47.1 (-48.4, 142.6) 0.334 | 57.0 (-16.9, 131.0) 0.131 |
| CHD history | | | |
| Yes | 0 | 0 | 0 |
| No | -86.1 (-157.8, -14.4) 0.019 | -17.1 (-80.8, 46.6) 0.599 | -51.2 (-97.6, -4.7) 0.031 |
| hyperlipidemia history | | | |
| Yes | 0 | 0 | 0 |
| No | -19.2 (-79.9, 41.5) 0.535 | 21.0 (-24.9, 66.8) 0.370 | 3.8 (-32.0, 39.5) 0.837 |
| age at first gestation | 2.5 (-5.2, 10.2) 0.521 | -1.8 (-7.8, 4.2) 0.546 | 0.3 (-4.4, 4.9) 0.911 |
| age at menopause | 0.5 (-2.0, 3.0) 0.685 | -0.6 (-3.1, 1.9) 0.644 | 0.0 (-1.7, 1.7) 0.972 |
| number of pregnancies | | | |
| 0 | 0 | 0 | 0 |
| 1 | 0 | 0 | 0 |
| 2 | -0.6 (-109.0, 107.8) 0.991 | 8.1 (-54.0, 70.1) 0.799 | -0.4 (-54.3, 53.4) 0.988 |
| 3 | 4.1 (-108.6, 116.8) 0.944 | -18.5 (-84.8, 47.8) 0.584 | -21.3 (-77.9, 35.2) 0.460 |
| 4 | -20.7 (-147.4, 106.0) 0.749 | -19.6 (-99.2, 60.1) 0.630 | -33.2 (-99.6, 33.2) 0.327 |
| 5 | 70.7 (-83.5, 225.0) 0.369 | -0.4 (-111.7, 110.9) 0.994 | 12.2 (-74.8, 99.2) 0.783 |
| 6 | 45.2 (-181.2, 271.6) 0.696 | 1.0 (-217.3, 219.2) 0.993 | 16.6 (-133.0, 166.2) 0.828 |
| 8 | -319.1 (-641.9, 3.7) 0.054 | 0 | -314.8 (-609.7, -19.8) 0.037 |
| smoking status | | | |
| No | 0 | 0 | 0 |
| Occasional smokers | 101.5 (-128.2, 331.3) 0.387 | -139.0 (-358.4, 80.4) 0.215 | 15.5 (-138.1, 169.2) 0.843 |
| Regular smokers | -34.0 (-288.0, 220.0) 0.793 | 36.8 (-96.1, 169.7) 0.587 | -15.6 (-129.8, 98.5) 0.789 |
| drinking status | | | |
| No | 0 | 0 | 0 |
| Occasional drinkers | -156.7 (-275.4, -38.1) 0.010 | 5.9 (-65.8, 77.6) 0.871 | -35.1 (-96.1, 25.8) 0.259 |

| | | | |
|---|---|---|---|
| **Regular drinkers** | 58.4 (-139.4, 256.1) 0.563 | -1.0 (-148.6, 146.5) 0.989 | 19.0 (-95.3, 133.4) 0.744 |
| **antihypertensive drug use** | | | |
| **Yes** | 0 | 0 | 0 |
| **No** | -51.6 (-120.9, 17.8) 0.146 | 16.2 (-43.5, 76.0) 0.594 | -13.0 (-57.1, 31.0) 0.562 |
| **TC** | 68.1 (-77.5, 213.7) 0.360 | -56.4 (-200.1, 87.3) 0.442 | 28.8 (-70.3, 127.9) 0.569 |
| **HDCP** | | | |
| **Yes** | 0 | 0 | 0 |
| **No** | -3.6 (-101.3, 94.2) 0.943 | -96.3 (-177.2, -15.4) 0.020 | -35.0 (-96.4, 26.4) 0.264 |
| **GDM** | | | |
| **Yes** | 0 | 0 | 0 |
| **No** | 169.8 (-429.0, 768.7) 0.579 | -30.2 (-382.5, 322.2) 0.867 | 82.2 (-215.2, 379.5) 0.588 |
| **Occupation** | | | |
| **Worker** | 0 | 0 | 0 |
| **Famer** | 210.6 (-442.6, 863.9) 0.528 | 0 | 115.4 (-347.6, 578.4) 0.625 |
| **Cadre** | -123.3 (-783.5, 536.9) 0.715 | 0 | 12.0 (-604.3, 628.3) 0.969 |
| **Office and technical, personnel, doctor, teacher** | -105.0 (-647.2, 437.2) 0.705 | 53.4 (-367.1, 473.9) 0.804 | 119.7 (-288.2, 527.5) 0.565 |
| **Service worker** | 0 | 0 | 0 |
| **Housewife** | -145.6 (-564.9, 273.7) 0.497 | -77.6 (-471.1, 315.8) 0.699 | -36.0 (-414.1, 342.0) 0.852 |
| **Unemployed** | -116.3 (-499.6, 267.0) 0.552 | -161.1 (-518.0, 195.8) 0.377 | -40.0 (-399.0, 319.1) 0.827 |
| **Other** | -82.6 (-555.7, 390.6) 0.732 | -65.3 (-503.2, 372.6) 0.770 | 16.1 (-385.0, 417.2) 0.937 |
| **Education** | | | |
| **Illiterate** | 0 | 0 | 0 |
| **Primary school** | 21.0 (-125.7, 167.6) 0.780 | 147.3 (15.8, 278.7) 0.028 | 71.4 (-24.0, 166.8) 0.143 |
| **Junior high school** | 55.2 (-91.2, 201.6) 0.460 | 99.6 (-26.9, 226.1) 0.123 | 58.1 (-35.5, 151.8) 0.224 |
| **Senior high school** | 58.2 (-96.5, 212.9) 0.462 | 82.6 (-45.7, 211.0) 0.207 | 51.1 (-45.6, 147.8) 0.300 |
| **College** | 33.9 (-127.6, 195.4) 0.681 | 79.5 (-51.8, 210.8) 0.236 | 39.9 (-59.3, 139.2) 0.431 |
| **Marriage status** | | | |
| **Married** | 0 | 0 | 0 |
| **Widowed** | 0 | 0 | 0 |
| **Separated** | 55.3 (-37.7, 148.3) 0.245 | -21.6 (-89.0, 45.7) 0.529 | 11.3 (-42.5, 65.2) 0.680 |
| **Divorced** | 0 | -263.7 (-767.4, 240.0) 0.305 | -296.2 (-806.5, 214.1) 0.255 |

Adjusted model II adjusted for adjusted model I + TC; hypertensive disorder complicating pregnancy (HDCP); gestational diabetes mellitus (GDM);

## Table S13. Logistical regression analysis between change in PWV as a category variable in diabetes subgroups in Model I

| Exposure | Diabetes Yes | Diabetes No | Total |
|---|---|---|---|
| | OR (95%CI) *P*-value | OR (95%CI) *P*-value | OR (95%CI) *P*-value |
| Age | 1.0 (0.9, 1.0) 0.238 | 1.0 (1.0, 1.1) 0.532 | 1.0 (1.0, 1.0) 0.909 |
| BMI | 0.9 (0.9, 1.0) 0.162 | 1.0 (1.0, 1.1) 0.507 | 1.0 (0.9, 1.0) 0.649 |
| SBP | 1.0 (1.0, 1.0) 0.059 | 1.0 (1.0, 1.0) 0.013 | 1.0 (1.0, 1.0) <0.001 |
| DBP | 1.0 (1.0, 1.0) 0.236 | 1.0 (1.0, 1.0) 0.813 | 1.0 (1.0, 1.0) 0.284 |
| TG | 0.9 (0.6, 1.2) 0.470 | 1.2 (1.0, 1.6) 0.085 | 1.1 (0.9, 1.3) 0.464 |
| GGT | 1.0 (1.0, 1.0) 0.190 | 1.0 (1.0, 1.0) 0.580 | 1.0 (1.0, 1.0) 0.267 |
| LDL_C | 0.7 (0.5, 1.0) 0.036 | 1.1 (0.8, 1.3) 0.656 | 0.9 (0.8, 1.1) 0.255 |
| HDL_C | 0.6 (0.3, 1.5) 0.279 | 1.2 (0.7, 2.2) 0.544 | 1.0 (0.6, 1.5) 0.833 |
| FBG | 0.9 (0.7, 1.1) 0.205 | 0.8 (0.5, 1.2) 0.270 | 0.9 (0.8, 1.1) 0.274 |
| PBG | 1.0 (0.9, 1.1) 0.566 | 1.0 (0.9, 1.2) 0.715 | 1.0 (1.0, 1.1) 0.599 |
| HbA1c | 1.1 (0.8, 1.5) 0.592 | 0.9 (0.5, 1.6) 0.626 | 1.0 (0.8, 1.4) 0.761 |
| The number of childbirths | | | |
| 1 | 1.0 | 1.0 | 1.0 |
| 2 | 3.5 (1.5, 7.9) 0.003 | 1.0 (0.6, 1.7) 0.910 | 1.4 (0.9, 2.2) 0.122 |
| 3 | 4.4 (1.3, 14.5) 0.015 | 1.0 (0.4, 2.4) 0.925 | 1.6 (0.8, 3.1) 0.198 |
| CHD history | | | |
| Yes | 1.0 | 1.0 | 1.0 |
| No | 0.8 (0.4, 1.4) 0.415 | 1.1 (0.6, 1.9) 0.845 | 0.9 (0.6, 1.3) 0.516 |
| hyperlipidemia history | | | |
| Yes | 1.0 | 1.0 | 1.0 |
| No | 0.7 (0.4, 1.1) 0.120 | 1.0 (0.6, 1.5) 0.877 | 0.8 (0.6, 1.1) 0.202 |
| age at first gestation | 1.0 (1.0, 1.1) 0.417 | 1.0 (1.0, 1.1) 0.706 | 1.0 (1.0, 1.1) 0.472 |
| age at menopause | 1.0 (1.0, 1.0) 0.946 | 1.0 (1.0, 1.0) 0.912 | 1.0 (1.0, 1.0) 0.867 |
| number of pregnancies | | | |
| 0 | 1.0 | 1.0 | 1.0 |
| 1 | 1.0 | 1.0 | 1.0 |
| 2 | 0.5 (0.2, 1.3) 0.146 | 1.1 (0.6, 1.9) 0.770 | 0.9 (0.5, 1.4) 0.526 |
| 3 | 0.5 (0.2, 1.5) 0.208 | 1.0 (0.5, 1.9) 0.968 | 0.8 (0.5, 1.3) 0.339 |
| 4 | 0.4 (0.1, 1.2) 0.109 | 0.8 (0.4, 1.8) 0.653 | 0.7 (0.4, 1.2) 0.192 |
| 5 | 0.9 (0.2, 3.4) 0.839 | 0.9 (0.3, 2.6) 0.821 | 1.1 (0.5, 2.4) 0.806 |
| 6 | 1.6 (0.2, 10.6) 0.636 | 1.8 (0.3, 11.8) 0.556 | 2.0 (0.6, 6.8) 0.290 |
| 8 | 0.0 (0.0, Inf) 0.984 | 1.0 | 0.0 (0.0, Inf) 0.978 |
| smoking status | | | |
| No | 1.0 | 1.0 | 1.0 |
| Occasional smokers | 1.6 (0.3, 10.1) 0.590 | 0.8 (0.1, 7.2) 0.817 | 1.4 (0.4, 5.1) 0.607 |
| Regular smokers | 1.4 (0.2, 10.2) 0.727 | 1.0 (0.3, 3.4) 0.986 | 0.9 (0.3, 2.5) 0.892 |
| drinking status | | | |
| No | 1.0 | 1.0 | 1.0 |
| Occasional drinkers | 0.6 (0.2, 2.0) 0.403 | 1.3 (0.7, 2.4) 0.446 | 1.0 (0.6, 1.8) 0.885 |

| | | | |
|---|---|---|---|
| **Regular drinkers** | 1.6 (0.3, 8.2) 0.599 | 0.7 (0.1, 3.4) 0.657 | 1.0 (0.3, 2.8) 0.972 |
| **antihypertensive drug use** | | | |
| **Yes** | 1.0 | 1.0 | 1.0 |
| **No** | 0.8 (0.4, 1.5) 0.535 | 1.1 (0.6, 2.0) 0.650 | 1.0 (0.6, 1.4) 0.818 |

Adjusted model I adjusted for age; BMI; SBP; DBP; TG; LDL-C; HDL-C; FBG; PBG; HbA1c; CHD history; hyperlipidemia history; age at first gestation; age at menopause; number of pregnancies; smoking status; drinking status; antihypertensive drug use; hypoglycemic drug use

**Table S14. Logistical regression analysis between change in PWV as a category variable in diabetes subgroups in Model II**

| Exposure | Diabetes Yes | Diabetes No | Total |
|---|---|---|---|
| | OR (95%CI) *P*-value | OR (95%CI) *P*-value | OR (95%CI) *P*-value |
| Age | 1.0 (0.9, 1.0) 0.398 | 1.0 (1.0, 1.1) 0.287 | 1.0 (1.0, 1.1) 0.689 |
| BMI | 0.9 (0.8, 1.0) 0.075 | 1.0 (1.0, 1.1) 0.523 | 1.0 (0.9, 1.0) 0.549 |
| SBP | 1.0 (1.0, 1.0) 0.016 | 1.0 (1.0, 1.0) 0.009 | 1.0 (1.0, 1.0) <0.001 |
| DBP | 1.0 (1.0, 1.1) 0.005 | 1.0 (1.0, 1.0) 0.815 | 1.0 (1.0, 1.0) 0.011 |
| TG | 0.7 (0.4, 1.2) 0.201 | 1.6 (1.0, 2.5) 0.065 | 1.1 (0.8, 1.5) 0.697 |
| GGT | 1.0 (1.0, 1.0) 0.137 | 1.0 (1.0, 1.0) 0.487 | 1.0 (1.0, 1.0) 0.230 |
| LDL_C | 0.3 (0.1, 1.5) 0.133 | 2.1 (0.5, 9.2) 0.322 | 0.8 (0.3, 2.1) 0.611 |
| HDL_C | 0.3 (0.1, 1.5) 0.136 | 2.2 (0.5, 9.2) 0.263 | 0.9 (0.3, 2.3) 0.766 |
| FBG | 0.9 (0.7, 1.1) 0.211 | 0.8 (0.5, 1.2) 0.318 | 0.9 (0.8, 1.1) 0.309 |
| PBG | 1.0 (0.9, 1.1) 0.683 | 1.1 (0.9, 1.2) 0.460 | 1.0 (1.0, 1.1) 0.611 |
| HbA1c | 1.1 (0.8, 1.5) 0.599 | 0.8 (0.4, 1.5) 0.499 | 1.0 (0.8, 1.4) 0.892 |
| The number of childbirths | | | |
| 1 | 1.0 | 1.0 | 1.0 |
| 2 | 3.1 (1.3, 7.2) 0.009 | 0.9 (0.5, 1.6) 0.781 | 1.4 (0.9, 2.1) 0.182 |
| 3 | 4.1 (1.2, 14.3) 0.028 | 0.8 (0.3, 2.1) 0.680 | 1.5 (0.8, 3.0) 0.239 |
| CHD history | | | |
| Yes | 1.0 | 1.0 | 1.0 |
| No | 0.8 (0.4, 1.6) 0.550 | 1.1 (0.6, 2.0) 0.765 | 0.9 (0.6, 1.4) 0.610 |
| hyperlipidemia history | | | |
| Yes | 1.0 | 1.0 | 1.0 |
| No | 0.6 (0.3, 1.1) 0.096 | 1.0 (0.6, 1.6) 0.990 | 0.8 (0.6, 1.2) 0.262 |
| age at first gestation | 1.0 (1.0, 1.1) 0.370 | 1.0 (1.0, 1.1) 0.779 | 1.0 (1.0, 1.1) 0.414 |
| age at menopause | 1.0 (1.0, 1.0) 0.973 | 1.0 (1.0, 1.0) 0.979 | 1.0 (1.0, 1.0) 0.938 |
| number of pregnancies | | | |
| 0 | 1.0 | 1.0 | 1.0 |
| 1 | 1.0 | 1.0 | 1.0 |
| 2 | 0.6 (0.2, 1.6) 0.285 | 1.0 (0.6, 1.8) 0.998 | 0.8 (0.5, 1.4) 0.490 |
| 3 | 0.5 (0.2, 1.5) 0.235 | 0.9 (0.5, 1.7) 0.806 | 0.7 (0.4, 1.2) 0.240 |
| 4 | 0.4 (0.1, 1.4) 0.160 | 0.8 (0.4, 1.7) 0.503 | 0.6 (0.3, 1.2) 0.141 |
| 5 | 1.4 (0.3, 5.9) 0.632 | 0.8 (0.3, 2.3) 0.656 | 1.1 (0.5, 2.4) 0.845 |
| 6 | 2.2 (0.3, 16.5) 0.434 | 2.2 (0.3, 15.9) 0.421 | 2.1 (0.6, 7.4) 0.256 |
| 8 | 0.0 (0.0, Inf) 0.989 | 1.0 | 0.0 (0.0, Inf) 0.978 |
| smoking status | | | |
| No | 1.0 | 1.0 | 1.0 |
| Occasional smokers | 1.7 (0.3, 11.7) 0.569 | 0.8 (0.1, 7.7) 0.840 | 1.5 (0.4, 5.5) 0.547 |
| Regular smokers | 2.3 (0.3, 20.1) 0.458 | 1.2 (0.3, 4.4) 0.786 | 1.0 (0.4, 2.9) 0.970 |
| drinking status | | | |

| | | | |
|---|---|---|---|
| No | 1.0 | 1.0 | 1.0 |
| Occasional drinkers | 0.5 (0.1, 1.8) 0.284 | 1.1 (0.6, 2.2) 0.702 | 1.0 (0.5, 1.7) 0.881 |
| Regular drinkers | 0.9 (0.1, 5.6) 0.922 | 0.6 (0.1, 3.4) 0.597 | 0.8 (0.3, 2.5) 0.703 |
| antihypertensive drug use | | | |
| Yes | 1.0 | 1.0 | 1.0 |
| No | 0.8 (0.4, 1.4) 0.383 | 1.2 (0.7, 2.1) 0.580 | 0.9 (0.6, 1.4) 0.697 |
| TC | 2.3 (0.5, 9.8) 0.271 | 0.5 (0.1, 2.1) 0.357 | 1.2 (0.5, 2.8) 0.754 |
| HDCP | | | |
| Yes | 1.0 | 1.0 | 1.0 |
| No | 1.0 (0.4, 2.5) 0.954 | 0.6 (0.3, 1.3) 0.238 | 0.8 (0.5, 1.5) 0.550 |
| GDM | | | |
| Yes | 1.0 | 1.0 | 1.0 |
| No | 4600932.3 (0.0, Inf) 0.995 | 0.4 (0.0, 7.8) 0.555 | 0.8 (0.1, 10.7) 0.898 |
| occupation | | | |
| Worker | 1.0 | 1.0 | 1.0 |
| Famer | 6017287.8 (0.0, Inf) 0.995 | 1.0 | 0.6 (0.0, 28.1) 0.768 |
| Cadre | 2713105.5 (0.0, Inf) 0.995 | 1.0 | 543754.7 (0.0, Inf) 0.988 |
| Office and technical personnel, doctor, teacher | 0.0 (0.0, Inf) 0.990 | 0.9 (0.0, 32.5) 0.960 | 0.2 (0.0, 6.6) 0.379 |
| Service worker | 1.0 | 1.0 | 1.0 |
| Housewife | 0.1 (0.0, 2.3) 0.129 | 0.3 (0.0, 9.8) 0.511 | 0.1 (0.0, 2.5) 0.163 |
| Unemployed | 0.0 (0.0, 1.1) 0.058 | 0.2 (0.0, 3.8) 0.271 | 0.1 (0.0, 1.9) 0.121 |
| Other | 0.1 (0.0, 4.5) 0.201 | 0.2 (0.0, 8.7) 0.382 | 0.1 (0.0, 2.5) 0.150 |
| Education | | | |
| Illiterate | 1.0 | 1.0 | 1.0 |
| Primary school | 1.5 (0.4, 6.6) 0.561 | 2.4 (0.6, 10.0) 0.229 | 1.6 (0.6, 4.1) 0.325 |
| Junior high school | 1.2 (0.3, 5.2) 0.788 | 1.6 (0.4, 6.6) 0.489 | 1.2 (0.5, 2.9) 0.764 |
| Senior high school | 1.7 (0.4, 8.2) 0.483 | 2.0 (0.5, 8.3) 0.320 | 1.5 (0.6, 3.8) 0.432 |
| College | 0.7 (0.1, 4.0) 0.727 | 2.0 (0.5, 8.2) 0.356 | 1.2 (0.4, 3.2) 0.733 |
| Marriage status | | | |
| Married | 1.0 | 1.0 | 1.0 |
| Widowed | 1.0 | 1.0 | 1.0 |
| Separated | 1.1 (0.4, 2.7) 0.883 | 1.0 (0.5, 2.0) 0.919 | 1.1 (0.6, 1.8) 0.823 |
| Divorced | 1.0 | 0.0 (0.0, Inf) 0.981 | 0.0 (0.0, Inf) 0.987 |

Adjusted model II adjusted for adjusted model I + TC; hypertensive disorder complicating pregnancy (HDCP); gestational diabetes mellitus (GDM);

## Table S15. Liner regression analysis between change in PWV as a continuous variable in diabetes subgroups in Model I

| Exposure | Diabetes Yes | Diabetes No | Total |
|---|---|---|---|
| Age | -5.5 (-14.7, 3.8) 0.246 | -10.1 (-16.9, -3.4) 0.004 | -7.7 (-13.1, -2.4) 0.005 |
| BMI | 0.6 (-10.9, 12.0) 0.924 | -0.3 (-7.5, 7.0) 0.940 | -0.9 (-6.9, 5.2) 0.781 |
| SBP | -5.4 (-7.6, -3.2) <0.001 | -3.9 (-5.6, -2.1) <0.001 | -4.9 (-6.2, -3.6) <0.001 |
| DBP | 2.0 (0.0, 4.0) 0.047 | -1.0 (-4.1, 2.2) 0.558 | 1.5 (0.0, 2.9) 0.047 |
| TG | -0.6 (-46.4, 45.1) 0.978 | 9.3 (-22.2, 40.8) 0.562 | 2.9 (-22.7, 28.4) 0.827 |
| GGT | -1.4 (-3.5, 0.7) 0.194 | 0.2 (-1.1, 1.5) 0.734 | -0.4 (-1.6, 0.7) 0.439 |
| LDL_C | -9.5 (-49.7, 30.6) 0.643 | -4.3 (-32.4, 23.9) 0.767 | -7.4 (-30.2, 15.4) 0.523 |
| HDL_C | 18.3 (-100.7, 137.3) 0.763 | -8.3 (-81.5, 65.0) 0.825 | -3.4 (-65.2, 58.4) 0.915 |
| FBG | -7.1 (-32.4, 18.2) 0.582 | -36.9 (-88.6, 14.8) 0.162 | -9.2 (-29.0, 10.6) 0.364 |
| PBG | 4.9 (-5.6, 15.5) 0.362 | 6.5 (-8.9, 21.9) 0.411 | 5.7 (-2.2, 13.7) 0.157 |
| HbA1c | -8.5 (-52.9, 35.8) 0.706 | -55.0 (-132.0, 22.0) 0.162 | -13.1 (-47.8, 21.5) 0.458 |
| The number of childbirths | | | |
| 1 | 0 | 0 | 0 |
| 2 | 130.3 (22.4, 238.1) 0.018 | 10.1 (-58.7, 78.9) 0.773 | 52.8 (-5.0, 110.6) 0.074 |
| 3 | 140.5 (-21.0, 302.1) 0.089 | 59.1 (-49.4, 167.5) 0.286 | 87.2 (-1.5, 175.9) 0.054 |
| CHD history | | | |
| Yes | 0 | 0 | 0 |
| No | -55.3 (-149.6, 39.0) 0.251 | -37.4 (-108.8, 34.1) 0.306 | -54.5 (-110.6, 1.6) 0.057 |
| hyperlipidemia history | | | |
| Yes | 0 | 0 | 0 |
| No | -13.6 (-91.5, 64.3) 0.732 | 3.4 (-48.1, 55.0) 0.896 | -9.2 (-51.7, 33.3) 0.671 |
| age at first gestation | 0.5 (-9.3, 10.4) 0.915 | -1.7 (-8.5, 5.1) 0.628 | -0.2 (-5.7, 5.3) 0.949 |
| age at menopause | 0.1 (-3.1, 3.4) 0.927 | -1.1 (-3.9, 1.8) 0.454 | -0.2 (-2.3, 1.8) 0.813 |
| number of pregnancies | | | |
| 0 | 0 | 0 | 0 |
| 1 | 0 | 0 | 0 |
| 2 | -50.0 (-190.3, 90.2) 0.485 | 25.2 (-46.2, 96.7) 0.489 | -1.3 (-66.2, 63.6) 0.968 |
| 3 | -109.8 (-255.2, 35.6) 0.140 | 13.5 (-62.5, 89.5) 0.728 | -34.5 (-102.6, 33.7) 0.322 |
| 4 | -88.5 (-252.5, 75.5) 0.291 | 46.7 (-44.9, 138.3) 0.318 | -5.7 (-85.9, 74.5) 0.889 |
| 5 | -49.7 (-248.5, 149.1) 0.624 | 41.3 (-85.0, 167.6) 0.522 | 13.3 (-91.4, 118.0) 0.804 |
| 6 | -36.6 (-334.1, 260.8) 0.809 | -48.0 (-295.4, 199.4) 0.704 | -10.1 (-191.2, 170.9) 0.913 |
| 8 | -307.3 (-734.0, 119.4) 0.159 | 0 | -248.8 (-608.1, 110.4) 0.175 |
| smoking status | | | |
| No | 0 | 0 | 0 |
| Occasional smokers | 171.3 (-128.3, 470.9) 0.263 | -52.1 (-305.9, 201.7) 0.688 | 96.0 (-90.7, 282.7) 0.314 |
| Regular smokers | -49.3 (-351.7, 253.1) 0.750 | 53.4 (-91.1, 197.9) 0.469 | 20.3 (-113.0, 153.6) 0.765 |

| | | | |
|---|---|---|---|
| drinking status | | | |
| No | 0 | 0 | 0 |
| Occasional drinkers | -50.9 (-200.5, 98.6) 0.505 | 21.9 (-59.9, 103.7) 0.601 | -4.1 (-77.0, 68.8) 0.913 |
| Regular drinkers | 62.3 (-190.9, 315.5) 0.630 | 6.8 (-160.1, 173.7) 0.936 | 18.3 (-119.1, 155.6) 0.795 |
| antihypertensive drug use | | | |
| Yes | 0 | 0 | 0 |
| No | -1.1 (-90.9, 88.7) 0.981 | 31.9 (-36.5, 100.2) 0.361 | 12.0 (-41.1, 65.1) 0.658 |

Adjusted model I adjusted for age; BMI; SBP; DBP; TG; GGT; LDL-C; HDL-C; FBG; PBG; HbA1c; CHD history; hyperlipidemia history; age at first gestation; age at menopause; number of pregnancies; smoking status; drinking status; antihypertensive drug use; hypoglycemic drug use

## Table S16. Liner regression analysis between change in PWV as a continuous variable in diabetes subgroups in Model II

| Exposure | Diabetes Yes | Diabetes No | Total |
|---|---|---|---|
| Age | -5.3 (-15.6, 5.1) 0.320 | -7.8 (-15.0, -0.6) 0.034 | -6.2 (-12.0, -0.3) 0.038 |
| BMI | -0.9 (-13.2, 11.5) 0.891 | -0.2 (-7.6, 7.3) 0.964 | -1.7 (-7.9, 4.5) 0.595 |
| SBP | -5.9 (-8.2, -3.6) <0.001 | -4.3 (-6.0, -2.5) <0.001 | -5.5 (-6.9, -4.2) <0.001 |
| DBP | 5.6 (1.9, 9.2) 0.003 | 0.1 (-3.3, 3.4) 0.964 | 4.4 (2.3, 6.5) <0.001 |
| TG | -19.1 (-91.3, 53.1) 0.604 | 46.3 (-10.0, 102.7) 0.108 | 6.5 (-35.6, 48.6) 0.763 |
| GGT | -1.7 (-3.8, 0.5) 0.127 | 0.1 (-1.2, 1.4) 0.861 | -0.6 (-1.7, 0.6) 0.337 |
| LDL_C | -104.8 (-317.3, 107.7) 0.334 | 114.3 (-65.2, 293.8) 0.212 | -10.1 (-141.4, 121.1) 0.880 |
| HDL_C | -61.4 (-285.1, 162.3) 0.591 | 104.0 (-65.6, 273.6) 0.230 | 0.8 (-128.1, 129.7) 0.990 |
| FBG | -8.3 (-34.2, 17.7) 0.532 | -21.4 (-74.2, 31.3) 0.426 | -8.2 (-28.2, 11.8) 0.422 |
| PBG | 4.8 (-6.3, 15.8) 0.401 | 10.9 (-4.9, 26.7) 0.176 | 6.5 (-1.5, 14.6) 0.112 |
| HbA1c | -9.1 (-55.1, 37.0) 0.700 | -61.5 (-139.5, 16.5) 0.123 | -16.6 (-51.7, 18.5) 0.355 |
| The number of childbirths | | | |
| 1 | 0 | 0 | 0 |
| 2 | 105.1 (-6.1, 216.3) 0.065 | 11.1 (-58.9, 81.2) 0.755 | 49.1 (-9.5, 107.6) 0.101 |
| 3 | 118.1 (-48.5, 284.7) 0.166 | 45.2 (-65.8, 156.2) 0.425 | 76.3 (-14.1, 166.7) 0.098 |
| CHD history | | | |
| Yes | 0 | 0 | 0 |
| No | -47.1 (-143.0, 48.8) 0.336 | -29.5 (-103.5, 44.5) 0.435 | -51.5 (-108.3, 5.3) 0.076 |
| hyperlipidemia history | | | |
| Yes | 0 | 0 | 0 |
| No | -10.3 (-91.7, 71.0) 0.803 | 5.7 (-47.5, 59.0) 0.833 | -10.8 (-54.4, 32.9) 0.629 |
| age at first gestation | 1.3 (-8.9, 11.6) 0.799 | -1.7 (-8.7, 5.3) 0.636 | 0.4 (-5.2, 6.1) 0.885 |
| age at menopause | -0.1 (-3.4, 3.2) 0.941 | -0.9 (-3.8, 2.0) 0.545 | -0.4 (-2.5, 1.6) 0.680 |
| number of pregnancies | | | |
| 0 | 0 | 0 | 0 |

| | | | |
|---|---|---|---|
| 1 | 0 | 0 | 0 |
| 2 | -22.7 (-167.9, 122.6) 0.760 | 13.0 (-59.1, 85.1) 0.724 | -3.4 (-69.3, 62.4) 0.919 |
| 3 | -98.6 (-248.8, 51.5) 0.199 | 0.4 (-76.6, 77.3) 0.993 | -39.3 (-108.4, 29.8) 0.265 |
| 4 | -68.8 (-238.5, 100.9) 0.427 | 30.0 (-62.2, 122.3) 0.524 | -14.2 (-95.4, 67.0) 0.732 |
| 5 | -7.8 (-214.1, 198.6) 0.941 | 28.5 (-100.8, 157.8) 0.666 | 10.6 (-95.8, 116.9) 0.846 |
| 6 | -15.6 (-318.9, 287.6) 0.920 | -24.0 (-277.7, 229.7) 0.853 | -10.1 (-192.9, 172.7) 0.914 |
| 8 | -312.1 (-744.8, 120.5) 0.158 | 0 | -280.0 (-640.4, 80.5) 0.128 |
| smoking status | | | |
| No | 0 | 0 | 0 |
| Occasional smokers | 151.8 (-156.0, 459.6) 0.334 | -58.3 (-313.0, 196.4) 0.654 | 93.9 (-93.8, 281.6) 0.327 |
| Regular smokers | 56.7 (-283.4, 396.8) 0.744 | 86.7 (-67.6, 241.0) 0.271 | 34.6 (-104.8, 174.0) 0.627 |
| drinking status | | | |
| No | 0 | 0 | 0 |
| Occasional drinkers | -81.4 (-240.0, 77.2) 0.315 | 11.8 (-71.6, 95.1) 0.782 | -11.3 (-85.8, 63.2) 0.766 |
| Regular drinkers | 25.9 (-239.1, 290.8) 0.848 | -11.2 (-182.7, 160.4) 0.898 | -3.9 (-143.6, 135.8) 0.956 |
| antihypertensive drug use | | | |
| Yes | 0 | 0 | 0 |
| No | -9.1 (-101.8, 83.6) 0.848 | 37.6 (-31.7, 107.0) 0.288 | 8.2 (-45.6, 62.0) 0.765 |
| TC | 82.3 (-112.8, 277.4) 0.409 | -110.7 (-277.5, 56.1) 0.194 | -0.5 (-121.6, 120.6) 0.993 |
| HDCP | | | |
| Yes | 0 | 0 | 0 |
| No | 116.4 (-13.2, 246.0) 0.079 | -35.8 (-129.3, 57.8) 0.454 | 46.4 (-28.1, 121.0) 0.223 |
| GDM | | | |
| Yes | 0 | 0 | 0 |
| No | 417.5 (-384.2, 1219.1) 0.308 | -112.8 (-522.2, 296.5) 0.589 | 51.5 (-311.9, 415.0) 0.781 |
| Occupation | | | |
| Worker | 0 | 0 | 0 |
| Farmer | 44.1 (-830.9, 919.2) 0.921 | 0 | 38.6 (-527.2, 604.5) 0.894 |
| Cadre | -65.8 (-950.5, 818.9) 0.884 | 0 | 61.5 (-691.7, 814.8) 0.873 |
| Office and technical personnel, doctor, teacher | -312.1 (-1038.1, 413.8) 0.400 | 28.6 (-460.2, 517.4) 0.909 | -14.7 (-513.0, 483.6) 0.954 |
| Service worker | 0 | 0 | 0 |
| Housewife | -331.4 (-892.5, 229.8) 0.248 | -147.0 (-604.2, 310.2) 0.529 | -195.2 (-656.9, 266.6) 0.408 |
| Unemployed | -249.3 (-762.5, 264.0) 0.342 | -164.1 (-579.0, 250.8) 0.439 | -143.8 (-582.5, 294.8) 0.521 |
| Other | -206.6 (-840.4, 427.2) 0.523 | -189.6 (-698.3, 319.1) 0.465 | -154.8 (-644.7, 335.1) 0.536 |
| Education | | | |
| Illiterate | 0 | 0 | 0 |
| Primary school | 29.5 (-167.0, 226.0) 0.769 | 100.7 (-51.9, 253.3) 0.196 | 41.0 (-75.5, 157.6) 0.490 |
| Junior high school | -9.1 (-205.0, 186.9) 0.928 | 77.6 (-69.4, 224.7) 0.301 | 11.7 (-102.7, 126.1) 0.841 |
| Senior high school | 8.5 (-198.7, 215.7) 0.936 | 57.3 (-91.8, 206.5) 0.451 | 7.1 (-111.0, 125.2) 0.906 |
| College | -0.4 (-216.8, 216.0) 0.997 | 58.8 (-93.8, 211.3) 0.451 | 1.2 (-120.0, 122.5) 0.984 |

| | | | |
|---|---|---|---|
| **Marriage status** | | | |
| **Married** | 0 | 0 | 0 |
| **Widowed** | 0 | 0 | 0 |
| **Separated** | 60.1 (-64.6, 184.8) 0.345 | -8.0 (-86.2, 70.2) 0.841 | 24.6 (-41.2, 90.4) 0.464 |
| **Divorced** | 0 | -258.9 (-844.5, 326.6) 0.386 | -312.4 (-936.1, 311.4) 0.327 |

Adjusted model II adjusted for adjusted model I + TC; hypertensive disorder complicating pregnancy (HDCP); gestational diabetes mellitus (GDM)

| **Exposure** | N | % | Non-adjusted | Adjust I | Adjust II |
|---|---|---|---|---|---|
| | | | OR (95%CI) *P*-value | OR (95%CI) *P*-value | OR (95%CI) *P*-value |
| **PWV (cm/s) Category** | | | | | |
| **The number of childbirths** | | | | | |
| **1** | 527 | 24.1 | 1.0 | 1.0 | 1.0 |
| **2** | 383 | 26.4 | 1.2 (0.9, 1.6) 0.282 | 1.4 (0.9, 2.2) 0.107 | 1.4 (0.9, 2.2) 0.124 |
| **≥3** | 249 | 25.7 | 1.2 (0.8, 1.7) 0.395 | 1.6 (0.8, 3.1) 0.189 | 1.6 (0.8, 3.1) 0.195 |
| **P for trend** | | | 0.346 | 0.179 | 0.177 |
| **Continuous PWV (cm/s)** | | | | | |
| **The number of childbirths** | | | | | |
| **1** | 527 | 24.1 | 0 | 0 | 0 |
| **2** | 383 | 26.4 | -3.5 (-45.9, 38.9) 0.872 | 52.5 (-5.3, 110.3) 0.076 | 55.0 (-3.9, 113.8) 0.067 |
| **≥3** | 249 | 25.7 | -30.7 (-79.4, 18.1) 0.218 | 81.7 (-7.0, 170.5) 0.071 | 83.5 (-7.1, 174.1) 0.071 |
| **P for trend** | | | 0.256 | 0.058 | 0.056 |

Non-adjusted model adjusted for nothing

Adjusted model I adjusted for age; BMI; SBP; DBP; TG; GGT; LDL-C; HDL-C; FBG; PBG; HbA1c ; CHD history; hyperlipidemia history; age at first gestation; age at menopause; number of pregnancies; smoking status; drinking status; antihypertensive drug use; hypoglycemic drug use

Adjusted model II adjusted for adjusted model I + TC; hypertensive disorder complicating pregnancy (HDCP); gestational diabetes mellitus (GDM)
